# Association Between Food and Drug Administration Approval and Disparities in Immunotherapy Use Among Patients With Cancer in the US

**DOI:** 10.1001/jamanetworkopen.2022.19535

**Published:** 2022-06-30

**Authors:** Theresa Ermer, Maureen E. Canavan, Richard C. Maduka, Andrew X. Li, Michelle C. Salazar, Michael F. Kaminski, Matthew D. Pichert, Peter L. Zhan, Vincent Mase, Harriet Kluger, Daniel J. Boffa

**Affiliations:** 1 Section of Thoracic Surgery, Department of Surgery, Yale School of Medicine, New Haven, Connecticut; 2 Faculty of Epidemiology and Population Health, London School of Hygiene and Tropical Medicine, University of London, London, United Kingdom; 3 Faculty of Medicine, Friedrich-Alexander University Erlangen-Nürnberg, Erlangen, Germany; 4 Cancer Outcomes Public Policy and Effectiveness Research Center, Department of Internal Medicine, Yale School of Medicine, New Haven, Connecticut; 5 Department of Surgery, Yale School of Medicine, New Haven, Connecticut; 6 Yale School of Medicine, New Haven, Connecticut; 7 Section of Medical Oncology, Department of Internal Medicine, Yale School of Medicine, New Haven, Connecticut

## Abstract

**Importance:**

Clinical trials and compassionate use agreements provide selected patients with access to potentially life-saving treatments before approval by the Food and Drug Administration (FDA). Approval from the FDA decreases a number of access barriers; however, it is unknown whether FDA approval is associated with increases in the equitable use of novel therapies and reductions in disparities in use among patients with cancer in the US.

**Objective:**

To assess the association between FDA drug approval and disparities in the use of immunotherapy across health, sociodemographic, and socioeconomic strata before and after approval of the first checkpoint inhibitors for the treatment of patients with cancer in the US.

**Design, Setting, and Participants:**

This cohort study used data from the National Cancer Database to examine the use of immunotherapy across health, sociodemographic, and socioeconomic strata before and after FDA approval of the first checkpoint inhibitor therapies. A total of 402 689 patients 20 years or older who were diagnosed with stage IV non–small cell lung cancer (NSCLC), renal cell carcinoma (RCC), or melanoma of the skin between January 1, 2007, and December 31, 2018 (specific years varied by tumor type), were included.

**Exposures:**

Patient health (Charlson-Deyo comorbidity score and age), sociodemographic characteristics (sex, race, and ethnicity), and socioeconomic (insurance status and household income based on zip code of residence) characteristics.

**Main Outcomes and Measures:**

The association of patient characteristics with receipt of immunotherapy was evaluated in the 4 years before and the 3 years immediately after FDA approval using multivariable logistic regression modeling.

**Results:**

Among 402 689 patients (median [IQR] age, 68 [60-76 years]; 225 081 men [55.9%]), 347 233 had NSCLC, 43 714 had RCC, and 11 742 patients had melanoma. A total of 47 527 patients (11.8%) were Black, 15 763 (3.9%) were Hispanic, 375 874 (93.3%) were non-Hispanic, 335 833 (83.4%) were White, and 16 553 (4.1%) were of other races. Before FDA approval, 6271 patients (3.2%) with NSCLC, 1155 patients (4.8%) with RCC, and 504 patients (8.6%) with melanoma received immunotherapy compared with 23 908 patients (15.6%) with NSCLC, 3890 patients (19.7%) with RCC, and 1143 patients (19.3%) with melanoma after FDA approval. Before FDA approval, sociodemographic and socioeconomic characteristics were associated with variable immunotherapy administration by tumor type. For example, among those with NSCLC, Black patients were less likely to receive immunotherapy than White patients (odds ratio [OR], 0.78; 95% CI ,0.71-0.85; *P* < .001); among those with RCC, uninsured patients were less likely to receive immunotherapy than privately insured patients (OR, 0.31; 95% CI, 0.20-0.48; *P* < .001). After FDA approval, most disparities persisted, but several narrowed (eg, Black patients with NSCLC: OR, 0.87 [95% CI, 0.83-0.91; *P* < .001]; uninsured patients with RCC: OR, 0.60 [95% CI, 0.48-0.75; *P* < .001]). Although many disparities remained, some gaps across socioeconomic characteristics appeared to widen (eg, patients with NSCLC in the lowest vs highest income quartile: OR, 0.80; 95% CI, 0.76-0.83; *P* < .001), and new gaps emerged (eg, Black patients with RCC: OR, 0.82; 95% CI, 0.72-0.93; *P* = .003).

**Conclusions and Relevance:**

In this cohort study, disparities in immunotherapy use existed across a number of sociodemographic and socioeconomic characteristics among patients with NSCLC, RCC, and melanoma before FDA approval, including during the important period when clinical trials were accruing patients. Although FDA approval was associated with a significant increase in the use of immunotherapy, gaps persisted, suggesting that FDA approval may not eliminate disparities in the use of novel therapies.

## Key Points

**Question:**

Is drug approval by the Food and Drug Administration (FDA) associated with a reduction in disparities in novel cancer treatment use among patients with cancer in the US?

**Findings:**

In this cohort study of 402 689 patients with stage IV non–small cell lung cancer, renal cell carcinoma, and melanoma, factors such as Black race, Hispanic ethnicity, Medicaid insurance, lack of insurance, and lower household income were associated with significantly less frequent immunotherapy use before and after checkpoint inhibitors were approved by the FDA.

**Meaning:**

This study found that immunotherapy use in the US before and after FDA approval was heterogeneous, suggesting that FDA approval may narrow some gaps but not necessarily eliminate disparities in the use of novel therapies.

## Teaser

This cohort study uses data from the National Cancer Database to assess the association between drug approval by the Food and Drug Administration (FDA) and disparities in the use of immunotherapy among patients with stage IV non–small cell lung cancer, renal cell carcinoma, and melanoma in the US.

## Introduction

Over the past several decades, innovative cancer treatments have substantially improved the outlook for many patients with advanced-stage disease. In the US, a series of preclinical and clinical trials is required to establish the safety and efficacy of a novel treatment, a process that, ideally, ends with approval by the Food and Drug Administration (FDA). Although this process typically takes approximately 7 years,^1,2^ opportunities exist to access experimental treatments before FDA approval (eg, through clinical trial participation, compassionate use, or other agreements).^3,4^ The FDA’s expanded access program, which grants selected patients access to experimental drugs and devices, receives 1800 applications annually, 99% of which are approved.^3^ As a result, increasing numbers of patients with cancer can attribute their survival to receiving an innovative treatment before FDA approval.^5,6^

Approval by the FDA has the potential to facilitate access to novel treatments. The number of hospitals offering the novel treatments can increase substantially, from several hundred to several thousand. Many financial and administrative barriers are eliminated (eg, the clinical trial consent process or early access applications), and general awareness increases (eg, through advertising), making it easier for health care professionals and patients to discuss and commence treatment.^3,7^

The introduction of the first checkpoint inhibitor therapies for advanced-stage cancers provided an opportunity to evaluate the association between FDA approval and patterns of administration of a highly effective line of novel therapies. For example, the median survival of patients with advanced melanoma has historically been 6 to 9 months.^8^ With the advent of checkpoint inhibitor therapies, the median survival has been reported to exceed 60 months.^9^ Immunotherapy has similarly improved the outcomes of patients with advanced-stage lung cancer and renal cell carcinoma (RCC).^5,6^

The National Cancer Database (NCDB) is a comprehensive hospital-based cancer registry that captures data on the care of approximately 70% of all patients diagnosed with cancer in the US, including the use of immunotherapy.^10^ We examined differences in immunotherapy use based on patient health, sociodemographic, and socioeconomic characteristics obtained from the NCDB before and several years after immune checkpoint inhibitors were approved by the FDA.

## Methods

### Data Source

The institutional review board of the Yale School of Medicine approved this study with a waiver of informed consent because of the use of deidentified data. This study followed the Strengthening the Reporting of Observational Studies in Epidemiology (STROBE) reporting guideline for cohort studies.

### Study Population

The 2018 NCDB participant user file^11,12^ was queried for patients 20 years or older who were diagnosed with invasive American Joint Commission on Cancer (AJCC) clinical stage IV non–small cell lung cancer (NSCLC), RCC, and melanoma of the skin from January 1, 2007, to December 31, 2018. Although multiple editions of the *AJCC Cancer Staging Manual* (with different nomenclature) were published within the study period, the factors associated with advanced disease remained similar (the full staging strategy is available in eMethods in the Supplement). Only patients with complete information on immunotherapy receipt were included. A total of 3533 patients were excluded, and a sensitivity analysis did not identify any clinically important differences between included and excluded patients.

### Data Elements

Patient health, sociodemographic, and socioeconomic characteristics were selected as independent exposure variables based on exploratory analysis and existing literature.^13,14,15^ The following individual-level independent variables were included: comorbidity measured by a modified Charlson-Deyo comorbidity score (0 indicates no comorbid conditions recorded; 1 and ≥2 indicate an increasing number or severity of comorbid conditions^12^), age group (≤55 years, 56-65 years, 66-75 years, or >75 years), sex (male or female), race (Black, White, or other race), ethnicity (Hispanic or non-Hispanic), insurance status (none, private, Medicaid, Medicare, or other government insurance), and tumor histological characteristics (for NSCLC: adenocarcinoma, large cell carcinoma, NSCLC not otherwise specified, squamous cell carcinoma, and other [including NSCLC not further defined]; for RCC: clear cell carcinoma, papillary carcinoma, RCC not otherwise specified, and other [including RCC not further defined]; and for melanoma: acral lentiginous melanoma, cutaneous melanoma, melanoma not otherwise specified, and other [including melanoma of the skin not further defined]). The categorization of age was based on approximate quartiles to ensure the same category cutoffs for all 3 cancers. Other races were based on categories defined by the NCDB^12^ and included American Indian, Aleutian, or Eskimo; Asian Indian; Asian Indian or Pakistani, no other specification; Chamorran; Chinese; Fiji Islander; Filipino; Guamanian, no other specification; Hawaiian; Hmong; Japanese; Kampuchean (including Khmer and Cambodian); Korean; Laotian; Melanesian, no other specification; Micronesian, no other specification; New Guinean; Oriental, no other specification; other Asian, including Asian, no other specification; Pacific Islander, no other specification; Pakistani; Polynesian, no other specification; Samoan; Tahitian; Thai; Tongan; Vietnamese; and other. Quartiles of median household income (quartile 1, <$38 000; quartile 2, $38 000-$47 999; quartile 3, $48 000-$62 999; or quartile 4, ≥$63 000) and educational level (<7.0%, 7.0%-12.9%, 13.0%-20.9%, or ≥21.0% of people without a high school diploma) were estimates based on the patients’ zip code of residence.^12^

The primary outcome was the administration of immunotherapy. The study was framed around the dates of immune checkpoint inhibitor clinical trials, but the NCDB does not distinguish types of immunotherapy (see eMethods in the Supplement).

Disparities in health care were defined as scenarios in which a cohort of patients lacked an equitable opportunity to achieve their optimal health outcome.^16^ In the context of this study, the administration of immunotherapy as a treatment innovation was considered the opportunity for an optimal health outcome. Disparities were examined across sociodemographic and socioeconomic strata. Other independent variables, such as tumor histological characteristics and patient comorbidities, were examined in models but not considered as disparities.

#### Stratification by FDA Approval Dates for Immunotherapies

The study was stratified around FDA approval dates of the first checkpoint inhibitors to characterize factors associated with immunotherapy use in the time before and after FDA approval because these 2 periods offered distinctly different routes of accessing immunotherapy. In general, the preapproval era was defined as the 4 years before the year of FDA approval, and the early postapproval era was defined as the 3 years immediately after the year in which FDA approval occurred. The eras were categorized as follows: (1) for NSCLC, the preapproval era was January 1, 2011, to December 31, 2014, and the early postapproval era was January 1, 2015, to December 31, 2017^17,18^; (2) for melanoma, the preapproval era was January 1, 2007, to December 31, 2010, and the early postapproval era was January 1, 2011, to December 31, 2013^19^; and (3) for RCC, the preapproval era was January 1, 2012, to December 31, 2015, and the early postapproval era was January 1, 2016, to December 31, 2018.^20^ Additional details are available in eMethods in the Supplement.

### Statistical Analysis

Descriptive analysis of the distribution of immunotherapy receipt by covariates was conducted during the pre– and post–FDA approval eras. In addition, the characteristics of facilities providing immunotherapy in both eras were compared between cancer types. Statistically significant differences in these distributions were identified using the χ^2^ test or the Fisher exact test for categorical variables and the *t* test or the Mann-Whitney *U* test for continuous variables.

#### Missing Data Strategy

A survey performed to identify patterns in the missing data found that these data appeared to be missing at random. Therefore, multiple imputation via chained equations was used (eMethods in the Supplement).^21^ To create variance and pool effect estimates across imputed data sets, the Rubin rules were applied.^22^

#### Multivariable Logistic Regression Modeling

To identify factors independently associated with receipt of immunotherapy as a dichotomous outcome variable, multivariable logistic regression models were created. The models were stratified by period relative to FDA approval to evaluate the different profiles in the different periods. The primary descriptive model included the Charlson-Deyo comorbidity score, age, sex, race, ethnicity, insurance status, household income, and tumor histological characteristics as exposure variables. Educational level was noted to be correlated with household income (*r* = 0.67; *P* < .001) and was therefore excluded from our models. Adding an interaction term for age and Medicare insurance did not substantially change the results. We did not adjust for facility characteristics or receipt of other treatments (eg, chemotherapy, radiotherapy, or surgical procedures) in our primary analysis because this study was a patient-level analysis that focused on social factors associated with care (ie, we did not want to obscure outcomes for any patient cohort that was disproportionately cared for by hospitals that were less likely to administer immunotherapy). Data from sensitivity analyses including hospital- or treatment-related variables are available upon request. A clustering term for hospitals was added to all adjusted models.

#### Evaluation of Race, Ethnicity, and Socioeconomic Factors

To focus on race and ethnicity, we performed separate logistic regression analyses with modifications. The association of FDA approval with the use of immunotherapy was evaluated using a pre-post design, with 1 indicator variable per model for the pre– and post–FDA approval eras. Because race and ethnicity are associated with insurance status and household income,^23,24^ separate adjusted models were used for race and ethnicity as well as insurance status and income.

All statistical tests were 2-sided, with *P* = .05 as the threshold for statistical significance. All analyses were performed using SAS software, version 9.4 (SAS Institute Inc).

## Results

### Patient Characteristics

Overall, 402 689 eligible patients were identified, including 347 233 patients with NSCLC, 43 714 patients with RCC, and 11 742 patients with melanoma (eFigure in the Supplement). For the overall study population, the median (IQR) age was 68 (60-76) years and was similar across tumor types; 225 081 patients (55.9%) were male, and 177 608 (44.1%) were female (Table 1). With regard to race and ethnicity, 47 527 patients (11.8%) were Black, 15 763 (3.9%) were Hispanic, 375 874 (93.3%) were non-Hispanic, 335 833 (83.4%) were White, and 16 553 (4.1%) were of other races (including American Indian, Aleutian, or Eskimo; Asian Indian; Asian Indian or Pakistani, no other specification; Chamorran; Chinese; Fiji Islander; Filipino; Guamanian, no other specification; Hawaiian; Hmong; Japanese; Kampuchean [including Khmer and Cambodian]; Korean; Laotian; Melanesian, no other specification; Micronesian, no other specification; New Guinean; Oriental, no other specification; other Asian, including Asian, no other specification; Pacific Islander, no other specification; Pakistani; Polynesian, no other specification; Samoan; Tahitian; Thai; Tongan; Vietnamese; and other). Other patient characteristics differed significantly by cancer type (eg, Charlson-Deyo comorbidity score of 0: 116 892 patients [60.4%] with NSCLC vs 16 537 [69.0%] with RCC vs 4459 [83.7%] with melanoma; *P* < .001 for all comparisons) (eTables 1-4 in the Supplement).

**Table 1.** Patient Characteristics in the Pre–FDA Approval vs Early Post–FDA Approval Era Stratified by Receipt of Immunotherapy

Characteristic	Pre–FDA approval^a^			Early post–FDA approval^a^		
	Total patients, No.	Received immunotherapy, No. (%)^b^	*P* value^c^	Total patients, No.	Received immunotherapy, No. (%)^b^	*P* value^c^
Total, No.	223 337	7930 (3.6)	NA	179 352	28 941 (16.1)	NA
Cancer type						
NSCLC	193 546	6271 (3.2)	<.001	153 687	23 908 (15.6)	<.001
RCC	23 962	1155 (4.8)		19 752	3890 (19.7)	
Melanoma	5829	504 (8.6)		5913	1143 (19.3)	
Age, median (IQR), y	68 (60-76)	63 (56-70)	<.001	68 (60-76)	66 (58-73)	<.001
Age group, y						
≤55	35 549	1979 (5.6)	<.001	24 748	5024 (20.3)	<.001
56-65	60 351	2661 (4.4)		49 786	9077 (18.2)	
66-75	69 702	2411 (3.5)		57 562	9327 (16.2)	
>75	57 735	879 (1.5)		47 256	5513 (11.7)	
Sex						
Male	125 430	4445 (3.5)	.84	99 651	16 034 (16.1)	.55
Female	97 907	3485 (3.6)		79 701	12 907 (16.2)	
Race						
Black	26 405	727 (2.8)	<.001	21 122	2969 (14.1)	<.001
White	187 069	6831 (3.7)		148 764	24 569 (16.5)	
Other^d^	8304	316 (3.8)		8249	1214 (14.7)	
Missing	1559	56 (3.6)		1217	189 (15.5)	
Ethnicity						
Hispanic	8443	249 (2.9)	<.001	7320	1055 (14.4)	<.001
Non-Hispanic	207 483	7526 (3.6)		168 391	27 405 (16.3)	
Missing	7411	155 (2.1)		3641	481 (13.2)	
Insurance status						
Not insured	10 236	249 (2.4)	<.001	5649	684 (12.1)	<.001
Private	62 577	3428 (5.5)		48 982	9985 (20.4)	
Medicaid	17 760	589 (3.3)		15 522	2275 (14.7)	
Medicare	125 218	3389 (2.7)		103 642	15 239 (14.7)	
Other government	3306	100 (3.0)		3004	403 (13.4)	
Missing	4240	175 (4.1)		2553	355 (13.9)	
Household income quartile, $^e^						
<38 000	42 723	1250 (2.9)	<.001	30 969	4169 (13.5)	<.001
38 000-47 999	52 995	1711 (3.2)		39 181	5764 (14.7)	
48 000-62 999	57 903	2066 (3.6)		43 714	7157 (16.4)	
≥63 000	61 424	2444 (4.0)		46 578	8175 (17.6)	
Missing	8292	459 (5.5)		18 910	3676 (19.4)	
Charlson-Deyo comorbidity score						
0	137 888	5549 (4.0)	<.001	113 507	19 674 (17.3)	<.001
1	57 144	1811 (3.2)		38 697	5874 (15.2)	
≥2	28 305	570 (2.0)		27 148	3393 (12.5)	

Abbreviations: FDA, Food and Drug Administration; NA, not applicable; NSCLC, non–small cell lung cancer; RCC, renal cell carcinoma.

^a^ Included years vary by cancer type. In general, the preapproval era included the 4 years before FDA approval, and the postapproval era included the 3 years after FDA approval.

^b^ Percentages were calculated across rows (eg, of all included patients with NSCLC in the pre–FDA approval era, 3.2% received immunotherapy). Percentages might not total 100% due to rounding.

^c^ *P* value for comparison of patients who did and did not receive immunotherapy.

^d^ Other race included the following categories defined by the National Cancer Database^12^: American Indian, Aleutian, or Eskimo; Asian Indian; Asian Indian or Pakistani, no other specification; Chamorran; Chinese; Fiji Islander; Filipino; Guamanian, no other specification; Hawaiian; Hmong; Japanese; Kampuchean (including Khmer and Cambodian); Korean; Laotian; Melanesian, no other specification; Micronesian, no other specification; New Guinean; Oriental, no other specification; other Asian, including Asian, no other specification; Pacific Islander, no other specification; Pakistani; Polynesian, no other specification; Samoan; Tahitian; Thai; Tongan; Vietnamese; and other.

^e^ Quartiles based on median annual household income of people in the patient’s zip code of residence.

### Immunotherapy

**Figure 1.**
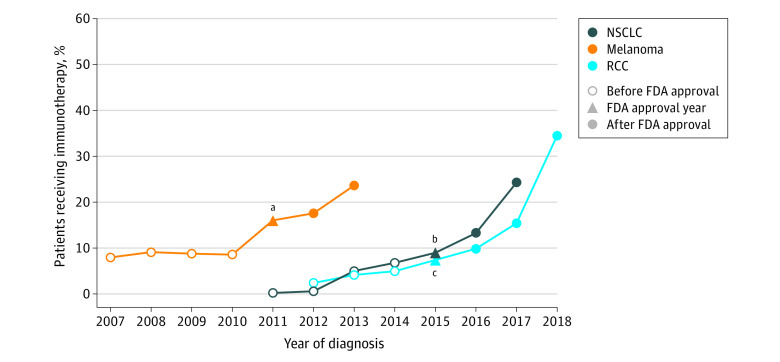
Receipt of Immunotherapy for Stage IV Cancer Over Time Among all patients who received immunotherapy within a diagnosis year. The Food and Drug Administration (FDA) approval year pertains to the year the first immune checkpoint inhibitor therapy for the respective cancer type was approved (eMethods in the Supplement). NSCLC indicates non–small cell lung cancer; RCC, renal cell carcinoma. ^a^For melanoma, the FDA approval year was included in the post–FDA approval era. The pre–FDA approval era was 2007 to 2010, and the early post–FDA approval era was 2011 to 2013. ^b^For NSCLC, the FDA approval year was included in the post–FDA approval era. The pre–FDA approval era was 2011 to 2014, and the early post–FDA approval era was 2015 to 2017. ^c^For RCC, the FDA approval year was included in the pre–FDA approval era. The pre–FDA approval era was 2012 to 2015, and the early post–FDA approval era was 2016 to 2018.

The frequency of immunotherapy use varied by tumor type over time (Figure 1). For each tumor type, there was a progressive increase in the use of immunotherapy leading up to FDA approval, then a substantial increase in use in the years after FDA approval. For example, immunotherapy use among patients with stage IV NSCLC was 0.2% in 2011, 9.0% in 2015, and 24.3% in 2017.

Among 223 337 patients in the preapproval era, 7930 (3.6%) received immunotherapy, including 6271 (3.2%) of 193 546 with NSCLC, 1155 (4.8%) of 23 962 with RCC, and 504 (8.6%) of 5829 with melanoma. In comparison, among 179 352 patients in the early postapproval era, 28 941 (16.1%) received immunotherapy, including 23 908 (15.6%) of 153 687 with NSCLC, 3890 (19.7%) of 19 752 with RCC, and 1143 (19.3%) of 5913 with melanoma. In general, compared with patients who did not receive immunotherapy in the preapproval (n = 215 407) and postapproval (n = 150 411) eras, those who received immunotherapy were healthier (eg, Charlson-Deyo comorbidity score of 0 in preapproval era: 5549 patients [70.0%] vs 132 339 patients [61.4%]; in postapproval era: 19 674 [68.0%] vs 93 833 [62.4%]), younger (eg, age ≤55 years in preapproval era: 1979 patients [25.0%] vs 33 570 patients [15.6%]; in postapproval era: 5024 patients [17.4%] vs 19 724 patients [13.1%]), lived in communities with higher household income (eg, ≥$63 000 in preapproval era: 2444 patients [30.8%] vs 58 980 patients [27.4%]; in postapproval era: 8175 patients [28.2%] vs 38 403 [25.5%]), and were more likely to be White (preapproval era: 6831 patients [86.1%] vs 180 238 patients [83.7%]; postapproval era: 24 569 patients [84.9%] vs 124 195 patients [82.6%]) compared with those who did not.

These unadjusted observations varied by cancer type (Table 1; eTables 1-4 in the Supplement). For example, in the preapproval era, the prevalence of immunotherapy use among patients with private insurance was 13.7% for those with melanoma but only 4.8% for those with NSCLC.

### Factors Associated With Immunotherapy Administration

Multivariable adjusted analyses were performed to identify factors associated with the use of immunotherapy. These analyses were stratified by FDA approval era.

#### Before FDA Approval

**Figure 2.**
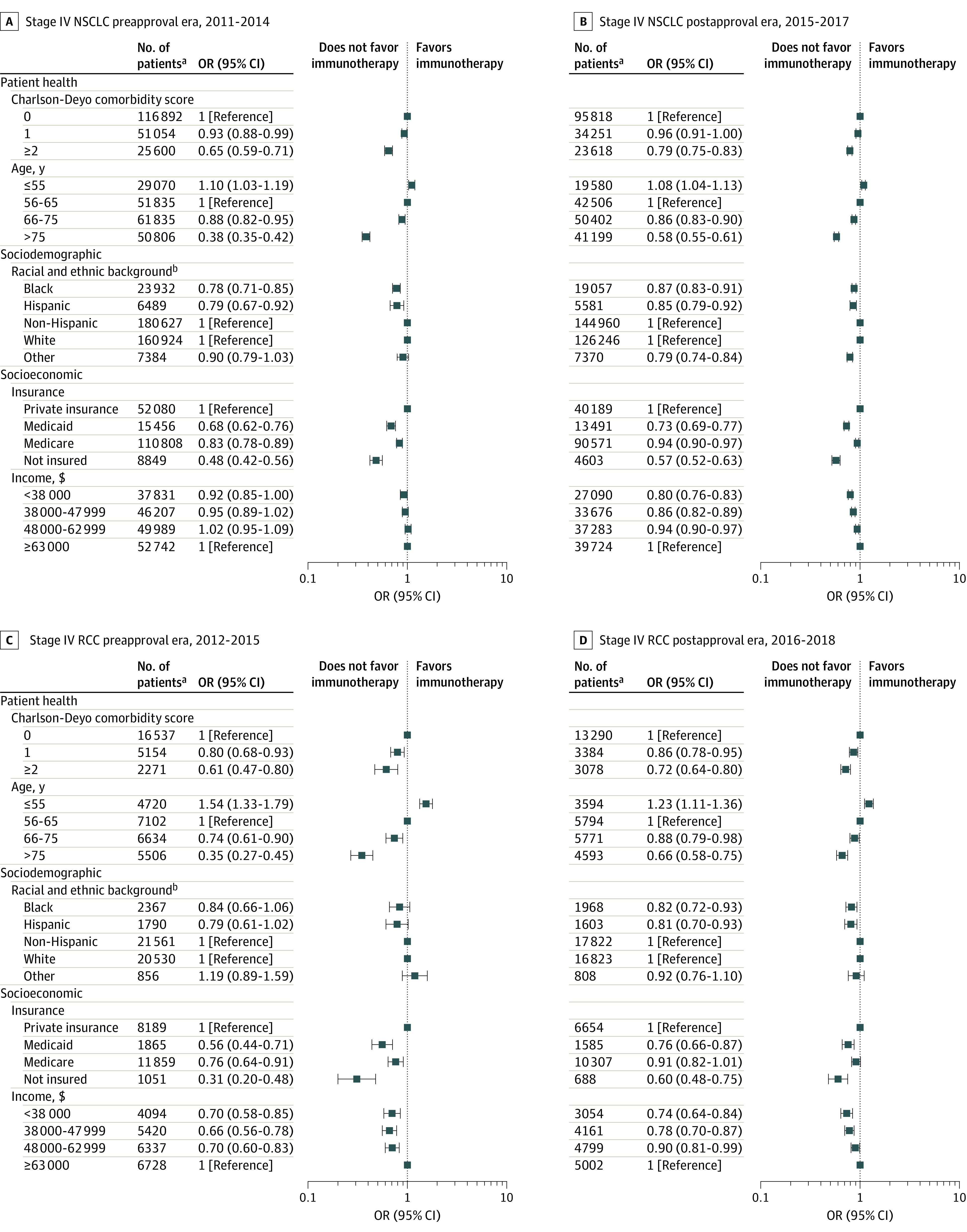
Receipt of Immunotherapy Among Patients With Non–Small Cell Lung Cancer (NSCLC) and Renal Cell Carcinoma (RCC) Data were derived from multivariable logistic regression models. Displayed covariates were selected from a larger multivariable logistic regression model that also included sex and tumor histological characteristics (additional details are available in eTables 4 and 5 in the Supplement). OR indicates odds ratio. ^a^Odds ratio estimates were generated based on 10 imputations with this sample. ^b^Non-Hispanic serves as the reference for Hispanic and White as the reference for Black and other. Although they are presented together, in the National Cancer Database^12^ and in our models, race and ethnicity were treated as separate variables. See Methods for details on other races and ethnicities.

**Figure 3.**
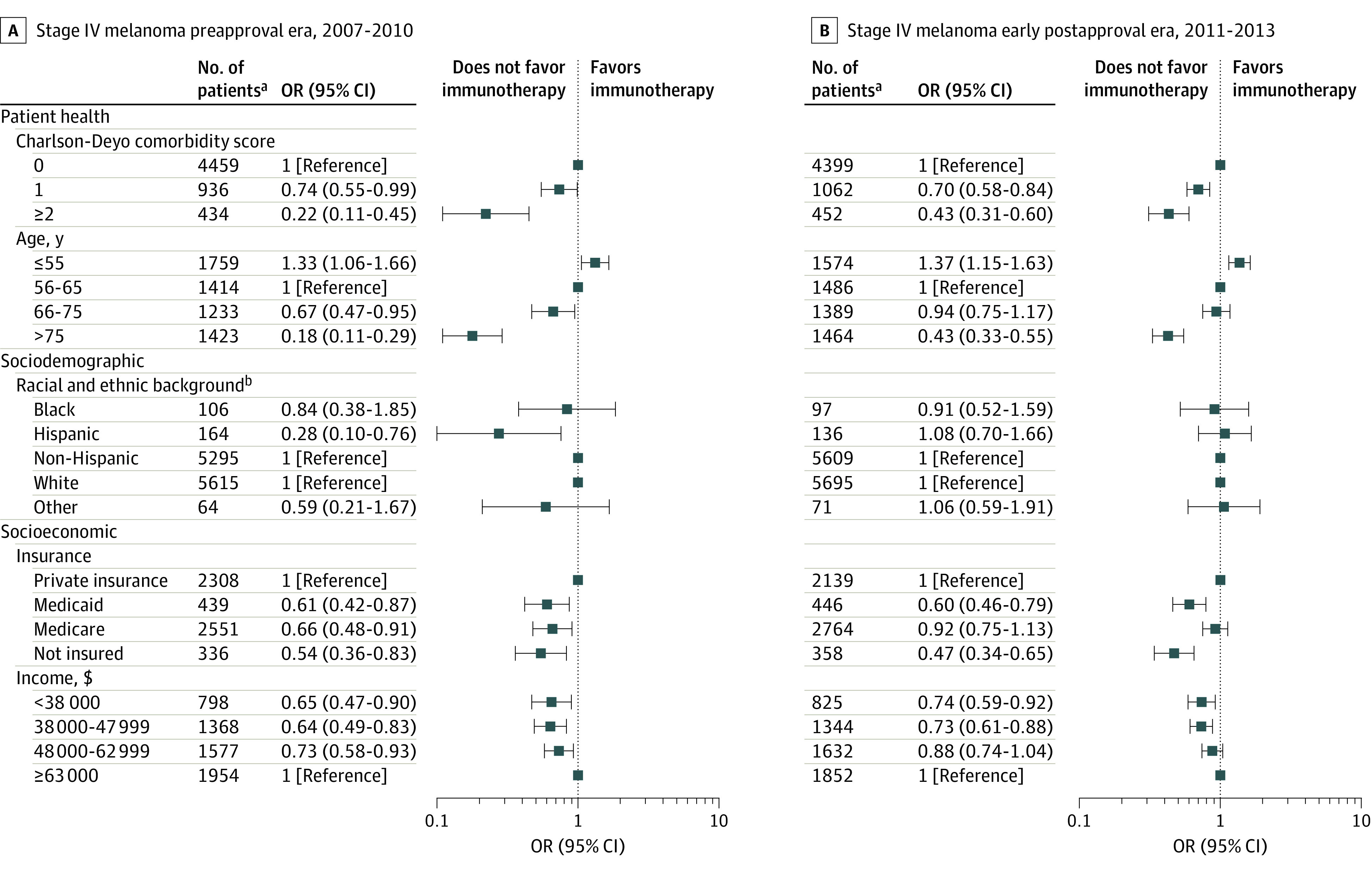
Receipt of Immunotherapy Among Patients With Melanoma Data were derived from multivariable logistic regression models. The displayed covariates were selected from a larger multivariable logistic regression model that also included sex and tumor histological characteristics (additional details available in eTable 7 in the Supplement). OR indicates odds ratio. ^a^Odds ratio estimates were generated based on 10 imputations with this sample. ^b^Non-Hispanic serves as the reference for Hispanic and White as the reference for Black and other. Although they are presented together, in the National Cancer Database^12^ and in our models, race and ethnicity were treated as separate variables. Other races include the following categories defined by the National Cancer Database^12^: American Indian, Aleutian, or Eskimo; Asian Indian; Asian Indian or Pakistani, no other specification; Chamorran; Chinese; Fiji Islander; Filipino; Guamanian, no other specification; Hawaiian; Hmong; Japanese; Kampuchean (including Khmer and Cambodian); Korean; Laotian; Melanesian, no other specification; Micronesian, no other specification; New Guinean; Oriental, no other specification; other Asian, including Asian, no other specification; Pacific Islander, no other specification; Pakistani; Polynesian, no other specification; Samoan; Tahitian; Thai; Tongan; Vietnamese; and other.

In the preapproval era, logistic regression models identified several factors associated with patient health, sociodemographic, and socioeconomic characteristics that were associated with immunotherapy use (Figure 2 and Figure 3; eTables 4-7 in the Supplement). In terms of health, the likelihood of receiving immunotherapy decreased incrementally among patients with a higher number of comorbidities (eg, among patients with NSCLC: odds ratio [OR], 0.93 [95% CI, 0.88-0.99; *P* = .03] for a Charlson-Deyo comorbidity score of 1 and 0.65 [95% CI, 0.59-0.71; *P* < .001] for a score of ≥2 compared with a score of 0) and patients who were older (eg, among patients with NSCLC: OR, 1.10 [95% CI, 1.03-1.19; *P* = .01] for age ≤55 years, 0.88 [95% CI, 0.82-0.95; *P* = .001] for age 66-75 years, and 0.38 [95% CI, 0.35-0.42; *P* < .001] for age >75 years compared with age 56-65 years) (Figure 2A).

Associations between sociodemographic characteristics and the use of immunotherapy varied by cancer type. Among patients with NSCLC, Black race was associated with lower likelihood of receiving immunotherapy compared with White race (OR, 0.78; 95% CI, 0.71-0.85; *P* < .001), whereas race was not associated with the likelihood of receiving immunotherapy among patients with RCC (eg, Black vs White race: OR, 0.84; 95% CI, 0.66-1.06; *P* = .14) and melanoma (eg, Black vs White race: OR, 0.84; 95% CI, 0.38-1.85; *P* = .66). Hispanic ethnicity was associated with a lower likelihood of receiving immunotherapy for NSCLC (OR, 0.79; 95% CI, 0.67-0.92; *P* = .003) and melanoma (OR, 0.28; 95% CI, 0.10-0.76; *P* = .01). A similar but nonsignificant pattern was observed among patients with RCC (OR, 0.79; 95% CI, 0.61-1.02; *P* = .07).

With respect to socioeconomic factors, patients who had no insurance, Medicare, or Medicaid were consistently less likely to receive immunotherapy. For example, uninsured patients with RCC were significantly less likely to receive immunotherapy than privately insured patients (OR, 0.31; 95% CI, 0.20-0.48; *P* < .001). Across all 3 types of cancer, patients with Medicare were less likely to receive immunotherapy (NSCLC: OR, 0.83 [95% CI, 0.78-0.89; *P* < .001]; RCC: OR, 0.76 [95% CI, 0.64-0.91; *P* = .004]; and melanoma: OR, 0.66 [95% CI, 0.48-0.91; *P* = .01]) compared with patients with private insurance. Patients with Medicaid were significantly less likely to receive immunotherapy than those with private insurance (eg, among those with RCC: OR, 0.56; 95% CI, 0.44-0.71; *P* < .001). Living within a community in a lower household income quartile was associated with a lower likelihood of receiving immunotherapy. For example, among patients with RCC and melanoma, the ORs for income quartiles 1 to 3 ranged between 0.64 (95% CI, 0.49-0.83; *P* = .001) for those with melanoma in quartile 2 and 0.73 (95% CI, 0.58-0.93; *P* = .01) for those with melanoma in quartile 3 and were all significantly lower compared with quartile 4 (eg, quartile 1: OR, 0.65 [95% CI, 0.47-0.90; *P* = .01] for melanoma and 0.70 [95% CI, 0.58-0.85; *P* < .001] for RCC). However, among patients with NSCLC, a significant association was observed only for those in quartile 1 (OR, 0.92; 95% CI, 0.85-1.00; *P* = .048).

#### After FDA Approval

After FDA approval, immunotherapy receipt increased substantially among patients with the 3 types of cancer examined (from 3.6% to 16.1%). In the early postapproval era, several factors associated with immunotherapy use were identified (eTables 4-6 in the Supplement). Similar to the preapproval era, receipt of immunotherapy in the postapproval era was significantly associated with patient health, sociodemographic, and socioeconomic characteristics. For example, patients 55 years or younger had a greater likelihood of receiving immunotherapy (NSCLC: OR, 1.08 [95% CI, 1.04-1.13; *P* < .001]; RCC: OR, 1.23 [95% CI, 1.11-1.36; *P* < .001]; and melanoma: OR, 1.37 [95% CI, 1.15-1.63; *P* < .001]) compared with patients aged 56 to 65 years (Figure 2B and D; Figure 3B). However, several factors identified in the preapproval era were no longer statistically significant in the multivariable models after FDA approval (eg, Hispanic vs non-Hispanic ethnicity among patients with melanoma: OR, 1.08 [95% CI, 0.70-1.66; *P* = .74]; Medicare vs private insurance among patients with melanoma: OR, 0.92 [95% CI, 0.75-1.13; *P* = .41]; Medicare vs private insurance among patients with RCC: OR, 0.91 [95% CI, 0.82-1.01; *P* = .07]).

Many differences persisted, some of which appeared to narrow (eg, Black patients with NSCLC: OR, 0.87 [95% CI, 0.83-0.91; *P* < .001] vs White patients; uninsured patients with RCC: OR, 0.60 [95% CI, 0.48-0.75; *P* < .001] vs patients with private insurance). Other variables were only associated with immunotherapy use in the early postapproval era. For example, Black race among patients with RCC was associated with a lower likelihood of receiving immunotherapy (OR, 0.82; 95% CI, 0.72-0.93; *P* = .003) compared with White race. Other examples of newly significant factors associated with immunotherapy use in the early postapproval era included Hispanic vs non-Hispanic ethnicity among patients with RCC (OR, 0.81 [95% CI, 0.70-0.93; *P* = .003] vs 0.79 [95% CI, 0.61-1.02; *P* = .07] in the preapproval era) and household income quartile 2 (OR, 0.86 [95% CI, 0.82-0.89; *P* < .001] vs 0.95 [95% CI, 0.89-1.02; *P* = .19] in the preapproval era) and quartile 3 (OR, 0.94 [95% CI, 0.90-0.97; *P* = .001] vs 1.02 [95% CI, 0.95-1.09; *P* = .62] in the preapproval era) vs quartile 4 among patients with NSCLC.

### FDA Approval and Immunotherapy Receipt by Race and Ethnicity

Separate models were created for race and ethnicity and socioeconomic characteristics using multivariable logistic regression analysis in a pre-post design for the 3 types of cancer (eTables 7-9 in the Supplement). The post–FDA approval era was associated with significantly increased immunotherapy use in all models. For example, in the race and ethnicity model , the ORs were 5.59 (95% CI, 5.43-5.75; *P* < .001) for patients with NSCLC, 5.06 (95% CI, 4.72-5.43; *P* < .001) for patients with RCC, and 2.66 (95% CI, 2.37-2.98; *P* < .001) for patients with melanoma. Black patients were less likely to receive immunotherapy for NSCLC (OR, 0.78; 95% CI, 0.75-0.81; *P* < .001) and RCC (OR, 0.74; 95% CI, 0.66-0.82; *P* < .001) compared with White patients, and Hispanic patients were less likely to receive immunotherapy for NSCLC (OR, 0.78; 95% CI, 0.73-0.84; *P* < .001), RCC (OR, 0.70; 95% CI, 0.62-0.79; *P* < .001), and melanoma (OR, 0.64; 95% CI, 0.43-0.93; *P* = .02) compared with non-Hispanic patients.

A number of socioeconomic factors were also associated with lower immunotherapy use. For example, uninsured patients continued to be less likely to receive immunotherapy for NSCLC (OR, 0.54; 95% CI, 0.50-0.58; *P* < .001), RCC (OR, 0.48; 95% CI, 0.39-0.58; *P* < .001), and melanoma (OR, 0.48; 95% CI, 0.37-0.61; *P* < .001) compared with privately insured patients.

### Facility Characteristics and Patient Travel in Preapproval Era

The proportion of hospitals in the NCDB that administered immunotherapy differed substantially by cancer type and FDA approval era. For example, in the preapproval era, 934 of 1259 hospitals (74.2%) that treated patients with stage IV NSCLC administered immunotherapy to at least 1 patient, whereas only 441 of 1255 hospitals (35.1%) that treated patients with RCC and 253 of 1031 hospitals (24.5%) that treated patients with melanoma administered immunotherapy to at least 1 patient (Table 2). Patients with RCC and melanoma who received immunotherapy were more frequently treated at academic hospitals (126 of 441 hospitals [28.6%] that treated at least 1 patient with RCC with immunotherapy and 71 of 253 hospitals [28.1%] that treated at least 1 patient with melanoma with immunotherapy) compared with patients with NSCLC (174 of 934 hospitals [18.6%] that treated at least 1 patient with NSCLC with immunotherapy). Patients with NSCLC were less likely to travel more than 1 hour to receive care ( travel >60 miles: 511 of 6271 patients [8.1%] with NSCLC vs 188 of 1155 patients [16.3%] with RCC and 105 of 504 patients [20.8%] with melanoma).

**Table 2.** Facility Characteristics and Patient Logistical Considerations in Pre–FDA Approval and Early Post–FDA Approval Eras

Characteristic	Facilities, No./total No. (%)					
	NSCLC^a^		RCC^b^		Melanoma^c^	
	Pre–FDA approval	Early post–FDA approval	Pre–FDA approval	Early post–FDA approval	Pre–FDA approval	Early post–FDA approval
Total facilities (any treatment), No.^d^	1259	1294	1255	1261	1031	1063
Facilities treating with immunotherapy^e^	934/1259 (74.2)	1243/1294 (96.1)	441/1255 (35.1)	927/1261 (73.5)	253/1031 (24.5)	440/1063 (41.4)
Facility type						
Community	153/934 (16.4)	263/1243 (21.1)	37/441 (8.4)	138/927 (14.9)	17/253 (6.7)	35/440 (8.0)
Comprehensive community program	373/934 (39.9)	476/1243 (38.3)	179/441 (40.6)	382/927 (41.2)	87/253 (34.4)	176/440 (40.0)
Academic	174/934 (18.6)	207/1243 (16.7)	126/441 (28.6)	192/927 (20.7)	71/253 (28.1)	113/440 (25.7)
Integrated network cancer program	232/934 (24.8)	296/1243 (23.8)	93/441 (21.1)	208/927 (22.4)	52/253 (20.6)	102/440 (23.2)
Unknown^f^	68/934 (7.3)	131/1243 (10.5)	36/441 (8.2)	77/927 (8.3)	59/253 (23.3)	67/440 (15.2)
Facility location						
Northeast	178/934 (19.1)	243/1243 (19.5)	85/441 (19.3)	172/927 (18.6)	43/253 (17.0)	75/440 (17.0)
Midwest	271/934 (29.0)	347/1243 (27.9)	119/441 (27.0)	257/927 (27.7)	66/253 (26.1)	125/440 (28.4)
South	336/934 (36.0)	431/1243 (34.7)	155/441 (35.1)	324/927 (35.0)	79/253 (31.2)	143/440 (32.5)
West	147/934 (15.7)	221/1243 (17.8)	76/441 (17.2)	167/927 (18.0)	39/253 (15.4)	83/440 (18.9)
Unknown^f^	68/934 (7.2)	131/1243 (10.5)	36/441 (8.2)	77/927 (8.3)	59/253 (23.3)	67/440 (15.2)
Patients who received immunotherapy during era, No.	6271	23 908	1155	3890	504	1143
Residential classification of patient receiving immunotherapy^g^						
Metropolitan	5010/6271 (80.0)	19 523/23 908 (81.7)	931/1155 (80.6)	3119/3890 (80.2)	427/504 (84.7)	923/1143 (80.8)
Urban	933/6271 (14.9)	3336/23 908 (14.0)	152/1155 (13.2)	573/3890 (14.7)	58/504 (11.5)	153/1143 (13.4)
Rural	149/6271 (2.4)	411/23 908 (1.7)	31/1155 (2.7)	83/3890 (2.1)	7/504 (1.4)	14/1143 (1.2)
Missing	179/6271 (2.9)	638/23 908 (2.7)	41/1155 (3.5)	115/3890 (3.0)	12/504 (2.4)	53/1143 (4.6)
Travel distance for patient receiving immunotherapy, miles^g^						
0-20	4109/6271 (65.5)	14 733/23 908 (61.6)	580/1155 (50.2)	2016/3890 (51.8)	246/504 (48.8)	605/1143 (52.9)
>20-40	994/6271 (15.9)	3311/23 908 (13.8)	173/1155 (15.0)	601/3890 (15.4)	96/504 (19.0)	185/1143 (16.2)
>40-60	356/6271 (5.7)	1239/23 908 (5.2)	84/1155 (7.3)	258/3890 (6.6)	42/504 (8.3)	86/1143 (7.5)
>60	511/6271 (8.1)	1597/23 908 (6.7)	188/1155 (16.3)	450/3890 (11.6)	105/504 (20.8)	200/1143 (17.5)
Missing	301/6271 (4.8)	3028/23 908 (12.7)	130/1155 (11.3)	565/3890 (14.5)	15/504 (3.0)	67/1143 (5.9)

Abbreviations: FDA, Food and Drug Administration; NSCLC, non–small cell lung cancer; RCC, renal cell carcinoma.

^a^ For NSCLC, the pre–FDA approval era was 2011 to 2014, and the early post–FDA approval era was 2015 to 2017.

^b^ For RCC, the pre–FDA approval era was 2012 to 2015, and the early post–FDA approval era was 2016 to 2018.

^c^ For melanoma, the pre–FDA approval era was 2007 to 2010, and the early post–FDA approval era was 2011 to 2013.

^d^ Includes facilities that treated at least 1 patient with specified stage IV cancers with any treatment during the pre–FDA approval era.

^e^ Includes facilities that treated at least 1 patient with specified stage IV cancers with immunotherapy during the pre–FDA approval era. These data include the number and percentage of total facilities providing any treatment in the respective era.

^f^ Includes facilities that treated at least 1 patient aged 0 to 39 years. Facility type and location were suppressed for patients aged 0 to 39 years because of small sample sizes. Therefore, some hospitals were counted twice, and the total does not equal the total number of facilities providing treatment with immunotherapy.

^g^ Includes the number and percentage of patients who received immunotherapy in the respective era.

In the postapproval era, more hospitals offered immunotherapy. The percentage administering immunotherapy increased to 1243 of 1294 hospitals (96.1%) that treated patients with NSCLC, 927 of 1261 hospitals (73.5%) that treated patients with RCC, and 440 of 1063 hospitals (41.4%) that treated patients with melanoma (Table 2). Compared with patients with NSCLC, those with RCC and melanoma continued to be more likely to receive treatment at academic institutions (192 of 927 hospitals [20.7%] that treated at least 1 patient with RCC with immunotherapy, and 113 of 440 hospitals [25.7%] that treated at least 1 patient with melanoma with immunotherapy compared with 207 of 1243 hospitals [16.7%] that treated at least 1 patient with NSCLC with immunotherapy) and to travel farther (>60 miles: 450 of 3890 patients [11.6%] with RCC and 200 of 1143 patients [17.5%] with melanoma vs 1597 of 23 908 patients [6.7%] with NSCLC).

## Discussion

This cohort study identified several scenarios in which Black and Hispanic patients were less likely to receive immunotherapy before the FDA approval era. Our findings of racial disparities in immunotherapy receipt before FDA approval are consistent with those of other studies that have examined patterns of immunotherapy use at varying points before FDA approval.^25,26,27^ However, to our knowledge, ethnic disparities in the use of immunotherapy in the US have not been previously described. Potential explanations for the current observations could include differences in the level of community engagement with medical innovations or recommendations from health care professionals.^7,28,29^ Implicit bias among clinicians has also been described and is commonly associated with the fear of inadequate adherence to prescribed treatment regimens and insufficient outpatient follow-up, increased efforts to provide patient education and adequate informed consent, and cultural disconnection resulting from lack of clinician diversity.^30^ Patients who speak only Spanish may encounter additional challenges because cancer care teams who speak only English may be less prepared or less willing to engage in nuanced shared decision-making conversations about novel treatments using interpretive services.^31,32,33,34,35^

In several of the scenarios assessed, socioeconomic characteristics (eg, insurance status) were associated with lower immunotherapy use before FDA approval. Insurance-associated differences in immunotherapy use have been previously described, albeit not specifically with regard to the preapproval era.^13,25,36,37^ Lower income has been associated with lower use of immunotherapy for melanoma^27,38^ but not for RCC and NSCLC. One possible explanation could be travel burden because, particularly among patients with RCC and melanoma, fewer than one-half of the hospitals that treated patients for the 3 cancer types included in this study were administering immunotherapy before FDA approval. Therefore, patients would likely have had to travel considerable distances to access immunotherapy, which is known to be particularly difficult for socioeconomically disadvantaged populations.^39^

The time frame leading up to FDA approval is an important period in which to observe disparities. The clinical trials that defined the safety and efficacy of these innovative treatments were accruing patients during this period and were likely a major route for patients in the preapproval era to access immunotherapy. As a result, disparities in the preapproval era likely reflect a lack of representativeness in the immunotherapy clinical trials.^16^ Several studies have found that Black and Hispanic patients are consistently underrepresented in clinical trials.^40,41^ For example, only 4% of participants in checkpoint inhibitor registration studies were Black or Hispanic, whereas Black and Hispanic individuals comprised 13.4% and 18.1% of the US population, respectively, during that period.^42^ Lack of inclusion of sufficient representative patients could have implications for the applicability of the findings because treatment safety and efficacy may vary across population groups.^43^ A recent example of the relevance of representativeness pertains to the low inclusion rates of minoritized populations in lung cancer screening studies, which prevented important differences in tobacco exposure from being identified.^44^ The lung cancer screening guidelines were recently revised to account for previously unrecognized differences across sociodemographic strata.^45^

After FDA approval, immunotherapy use increased substantially among patients with the 3 types of cancer examined (from 3.6% to 16.1%). However, although numerous gaps narrowed or even closed, many disparities persisted after FDA approval (eg, Black race among those with NSCLC and no insurance among those with RCC), and some new gaps emerged (eg, Black race and Hispanic ethnicity among those with RCC). These findings are consistent with those of previous, smaller studies investigating the post–FDA approval era.^38,46,47,48,49,50^

One explanation for the partial mitigation of disparities could be associated with increased patient access, given that the number of hospitals offering immunotherapy increased substantially from the pre- to postapproval period. This increase is consistent with previous studies describing rapid adoption of immunotherapy in the community after FDA approval.^48,51^ Direct-to-consumer checkpoint inhibitor advertising increased substantially during this period.^52,53^ Notably, commercial drug advertisements have been purported to incentivize minority groups to seek care more often than they incentivize nonminority groups.^53^

Persistent differences across patient populations could reflect unmitigated access barriers. For example, although FDA approval is generally followed by payer coverage, the extent and timing of coverage may differ across insurance plans, contractors, and regions, which could align with remaining disparities.^54,55,56,57^ More specifically, Medicare takes approximately 17 months after FDA approval to cover novel treatments nationally and often imposes requirements for previous authorization and step therapy,^54,55^ which could have implications for adoption among patients older than 64 years. We also observed a persistent association of socioeconomic characteristics with a lower likelihood of immunotherapy receipt, which is consistent with findings from most previous studies of patients with melanoma but had not been reported in studies involving patients with NSCLC and RCC.^27,37,38,58^ Many of the challenges outlined (eg, travel distance) had disproportionate consequences for socioeconomically disadvantaged patients and were likely incompletely mitigated by FDA approval.

### Limitations

This study has several limitations beyond those traditionally associated with observational research. First, the NCDB only captures first-line treatment from hospitals accredited by the Commission on Cancer. The distribution of facility and regional characteristics in this data set is therefore not generalizable to the general population,^59^ and some sociodemographic strata (eg, patients of Asian or Pacific Islander race or Hispanic ethnicity) are underrepresented.^10,11^ However, the NCDB’s overall case coverage has been calculated at 65% for lung cancer, 52% for melanoma, and 78% for kidney cancer,^10^ and the representation of Black and White patients has been almost equivalent in the past.^10,11^ Second, some patient subsets, such as Black patients with melanoma, were small, limiting the ability to fully understand race-associated disparities in this cohort.

Third, the NCDB does not capture specific medical information that could have implications for selection of patients for immunotherapy (eg, autoimmune disease, which is a current contraindication).^60^ Fourth, it is possible that the impact of FDA approval took longer to mitigate disparities than the period we examined. Fifth, this study was designed to evaluate differences in use, but the data in the NCDB are not appropriate for full exploration of the etiological factors associated with the observed disparities. More specifically, household income information is based on the median income of people in the zip code in which the patient resides and is not patient specific. Sixth, insurance plans can differ considerably by state, and we do not have state-level data. Some patients become eligible for Medicaid after being diagnosed with cancer; however, the NCDB only records insurance status once (at the time of diagnosis).^61,62,63,64^ Therefore, some patients’ insurance status may have been misclassified. Seventh, this study was designed to examine the direction, extent, and significance of differences in immunotherapy receipt, which is only the first phase in narrowing the observed gaps.

## Conclusions

This cohort study found that, during the important period leading up to FDA approval in which clinical trials were performed that defined the role of immunotherapy in the US, disparities in the use of immunotherapy among patients with NSCLC, RCC, and melanoma existed across socioeconomic and sociodemographic populations. Approval from the FDA was associated with significant increases in immunotherapy use; however, numerous differences across sociodemographic and socioeconomic strata remained, suggesting that FDA approval alone does not ensure the optimal administration of novel treatments in the US.

## Notes

Supplement. **eMethods.** Commission on Cancer Acknowledgment, Staging Strategy, NCDB Definition of Immunotherapy, Rationale Behind Selection of Study Periods, Missing Data Strategy, and Clinical Trial Variable **eFigure.** Patient Selection Steps **eTable 1.** Tumor Histological Characteristics **eTable 2.** Patient, Treatment, and Facility Characteristics for NSCLC, RCC, and Melanoma in the Pre–FDA Approval Era **eTable 3.** Patient, Treatment, and Facility Characteristics for NSCLC, RCC, and Melanoma in the Early Post–FDA Approval Era **eTable 4.** Multivariable Logistic Regression Models for NSCLC in the Pre–FDA Approval and Early Post–FDA Approval Eras **eTable 5.** Multivariable Logistic Regression Models for RCC in the Pre–FDA Approval and Early Post–FDA Approval Eras **eTable 6.** Multivariable Logistic Regression Models for Melanoma in the Pre–FDA Approval and Early Post–FDA Approval Eras **eTable 7.** Pre-Post Multivariable Logistic Regression Models for NSCLC Assessing Racial and Ethnic Disparities and Socioeconomic Disparities Separately **eTable 8.** Pre-Post Multivariable Logistic Regression Models for RCC Assessing Racial and Ethnic Disparities and Socioeconomic Disparities Separately **eTable 9.** Pre-Post Multivariable Logistic Regression Models for Melanoma Assessing Racial and Ethnic Disparities and Socioeconomic Disparities Separately
eReferences
