# Supplementary material for: Association Between Food and Drug Administration Approval and Disparities in Immunotherapy Use Among Patients With Cancer in the US
Source: JAMA Netw Open. 2022 Jun 30;5(6):e2219535. doi: 10.1001/jamanetworkopen.2022.19535 (PMC9247736; doi:10.1001/jamanetworkopen.2022.19535)
Supplement: Supplement. — eMethods. Commission on Cancer Acknowledgment, Staging Strategy, NCDB Definition of Immunotherapy, Rationale Behind Selection of Study Periods, Missing Data Strategy, and Clinical Trial Variable eFigure. Patient Selection Steps eTable 1. Tumor Histological Characteristics eTable 2. Patient, Treatment, and Facility Characteristics for NSCLC, RCC, and Melanoma in the Pre–FDA Approval Era eTable 3. Patient, Treatment, and Facility Characteristics for NSCLC, RCC, and Melanoma in the Early Post–FDA Approval Era eTable 4. Multivariable Logistic Regression Models for NSCLC in the Pre–FDA Approval and Early Post–FDA Approval Eras eTable 5. Multivariable Logistic Regression Models for RCC in the Pre–FDA Approval and Early Post–FDA Approval Eras eTable 6. Multivariable Logistic Regression Models for Melanoma in the Pre–FDA Approval and Early Post–FDA Approval Eras eTable 7. Pre-Post Multivariable Logistic Regression Models for NSCLC Assessing Racial and Ethnic Disparities and Socioeconomic Disparities Separately eTable 8. Pre-Post Multivariable Logistic Regression Models for RCC Assessing Racial and Ethnic Disparities and Socioeconomic Disparities Separately eTable 9. Pre-Post Multivariable Logistic Regression Models for Melanoma Assessing Racial and Ethnic Disparities and Socioeconomic Disparities Separately eReferences [file jamanetwopen-e2219535-s001.pdf]

Supplementary Online Content

Ermer T, Canavan ME, Maduka RC, et al. Association between Food and Drug Administration approval and disparities in immunotherapy use among patients with cancer in the US. *JAMA Netw Open*. 2022;5(6):e2219535. doi:10.1001/jamanetworkopen.2022.19535

eMethods. Commission on Cancer Acknowledgment, Staging Strategy, NCDB Definition of Immunotherapy, Rationale Behind Selection of Study Periods, Missing Data Strategy, and Clinical Trial Variable

eFigure. Patient Selection Steps

eTable 1. Tumor Histological Characteristics

eTable 2. Patient, Treatment, and Facility Characteristics for NSCLC, RCC, and Melanoma in the Pre–FDA Approval Era

eTable 3. Patient, Treatment, and Facility Characteristics for NSCLC, RCC, and Melanoma in the Early Post–FDA Approval Era

eTable 4. Multivariable Logistic Regression Models for NSCLC in the Pre–FDA Approval and Early Post–FDA Approval Eras

eTable 5. Multivariable Logistic Regression Models for RCC in the Pre–FDA Approval and Early Post–FDA Approval Eras

eTable 6. Multivariable Logistic Regression Models for Melanoma in the Pre–FDA Approval and Early Post–FDA Approval Eras

eTable 7. Pre-Post Multivariable Logistic Regression Models for NSCLC Assessing Racial and Ethnic Disparities and Socioeconomic Disparities Separately

eTable 8. Pre-Post Multivariable Logistic Regression Models for RCC Assessing Racial and Ethnic Disparities and Socioeconomic Disparities Separately

eTable 9. Pre-Post Multivariable Logistic Regression Models for Melanoma Assessing Racial and Ethnic Disparities and Socioeconomic Disparities Separately

eReferences

This supplementary material has been provided by the authors to give readers additional information about their work.

© 2022 Ermer T et al. *JAMA Network Open*.

eMethods. Commission on Cancer Acknowledgment, Staging Strategy, NCDB Definition of Immunotherapy, Rationale Behind Selection of Study Periods, Missing Data Strategy, and Clinical Trial Variable

Commission on Cancer Acknowledgment

The Commission on Cancer (CoC), the NCDB, and participating hospitals have not verified and are not responsible for the statistical validity of the data analysis or the conclusions derived by the authors.

Staging Strategy

For all included cases of NSCLC and RCC, disease stage was recorded according to the *AJCC Staging Manual* 7th Edition prior to 2018, and according to the 8th Edition from 2018. For melanoma of the skin, the study spanned the 6th and 7th editions of the *AJCC Staging Manual*. The staging criteria for stage IV RCC remained unchanged across the included time frame. Changes of the staging criteria for stage IV NSCLC and melanoma across the editions did not affect the classification of stage IV in our study and therefore no corrective measures were employed.

NCDB Definition of Immunotherapy

The NCDB datafield defines immunotherapy as any drug categorized as “Biologic therapy (Biologic response modifier, immunotherapy)” in the SEER*Rx Interactive Antineoplastic Drugs Database.[1,2] Overall, >400 drugs are classified as immunotherapy, including a range of interleukins, interferons, antiangiogenic drugs, tumor vaccines, monoclonal antibodies, and checkpoint inhibitors. The database also lists drugs that are currently only available in clinical trials.

In 2013, six drugs were reclassified from chemotherapy to immunotherapy: alemtuzumab, bevacizumab, rituximab, trastuzumab, pertuzumab, and cetuximab.

Rationale Behind Selection of Study Periods

The NCDB provides data as annual aggregates. The eras around FDA approval for the included cancers were determined relative to the following FDA approval dates:

- NSCLC - nivolumab for metastatic NSCLC (Mar 4, 2015 for *squamous* NSCLC[3] and Oct 9, 2015 for *non-squamous* NSCLC)[4] and pembrolizumab for metastatic NSCLC (Oct 2, 2015)[5]. Therefore, the “pre-approval era” was considered to be 2011 to 2014 and the “early post-approval era” was 2015 to 2017.
- RCC - nivolumab for metastatic RCC (Nov 23, 2015)[9]. Therefore, the “pre-approval era” was considered to be 2012 to 2015 and the “early post-approval era” was 2016 to 2018. Because the drug was approved at the end of 2015, the approval year was included in the pre-approval era.
- Melanoma - ipilimumab for metastatic melanoma (Mar 25, 2011)[6]. Therefore, the “pre-approval era” was considered to be 2007 to 2010 and the “early post-approval era” was 2011 to 2013. In 2014, pembrolizumab (Sep 4, 2014)[7] and nivolumab (Dec 22, 2014)[8] were FDA-approved for metastatic melanoma.

We limited the pre-FDA approval period in our study to the four years before the respective approval date for two reasons:

© 2022 Ermer T et al. *JAMA Network Open*.

1) Clinical stage reporting only became mandatory in the NCDB in 2008,[10] which is why >50% of staging information was missing (for melanoma) in early diagnosis years from 2004-2008.
2) The phase III trials of the first approved checkpoint inhibitors for NSCLC (NCT01295827 KEYNOTE-001)[11] and RCC (NCT01668784 CheckMate 025)[12] started in 2011 and 2012 respectively.

The clinical trial that led to approval of ipilimumab for melanoma started in 2004 (NCT00094653)[13]. However, to keep time frames consistent, we limited the pre-approval era for melanoma to the four years prior to FDA-approval, analogous to NSCLC and RCC.

For checkpoint inhibitors approved within the first 6 months of a calendar year the approval year was included in the *post*-approval era and when the FDA approval was in the second half of the calendar year the approval year was included in the *pre*-approval era. The early post-FDA approval era was limited to the first three years following FDA-approval as these were available for all three cancers.

Missing Data Strategy
Values were imputed for the following variables: race; ethnicity; insurance status; income; educational level; receipt of surgery, radiation, and chemotherapy; area of residence, and distance to treatment facility. In the NCDB, facility type and location are suppressed for all patients aged 39 years or younger to protect patient privacy. No patients aged 40 years or older had missing values for facility type and location. Therefore, facility type and location were not included as input variables in our multiple imputation model.

Clinical Trial Variable
Due to likely underreporting in the NCDB, clinical trial participation could not be verified.

© 2022 Ermer T et al. *JAMA Network Open*.

eFigure. Patient Selection Steps

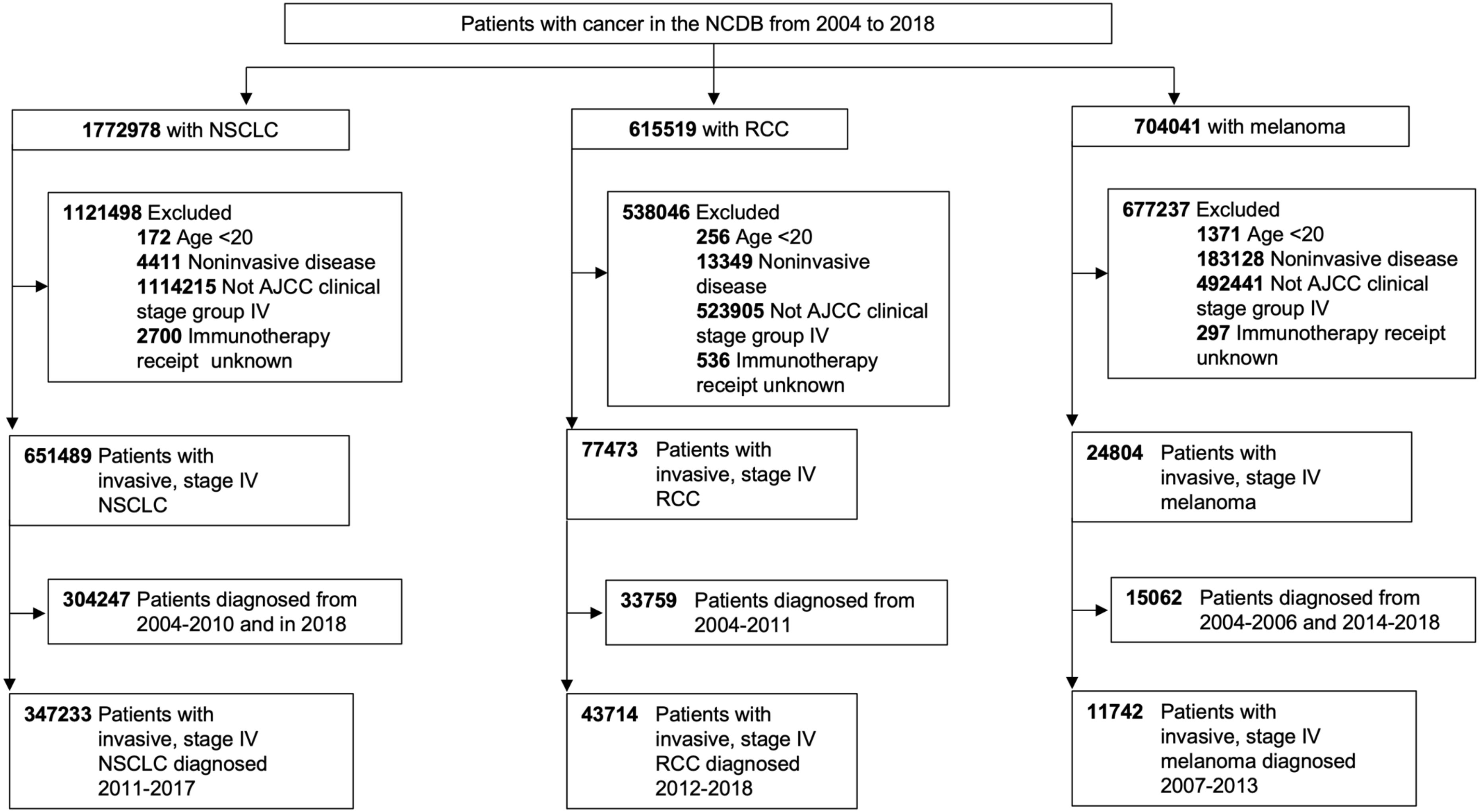

© 2022 Ermer T et al. *JAMA Network Open*.

eTable 1. Tumor Histological Characteristics

	Pre-FDA approval era[a]			Early post-FDA approval era[a]		
	All patients	Immunotherapy recipients[b]	P value	All patients	Immunotherapy recipients[b]	P value
**NSCLC**	**2011-2014**			**2015-2017**		
	**n = 193546**	**n = 6271**		**n = 153687**	**n = 23908**	
NSCLC, NOS[c]	21995	487 (2.2)		13406	1924 (14.4)	
SCC	38585	289 (0.8)		29750	3043 (10.2)	
Adenocarcinoma	111012	4939 (4.5)		93244	16938 (18.2)	
Large cell carcinoma	4380	86 (2)		2811	248 (8.8)	
Other[d]	17574	470 (2.7)	<.001	14476	1755 (12.1)	<.001
**RCC**	**2012-2015**			**2016-2018**		
	**n = 23962**	**n = 1155**		**n = 19752**	**n = 3890**	
Clear cell carcinoma	9527	699 (7.3)		8139	1922 (23.6)	
Papillary carcinoma	924	29 (3.1)		713	127 (17.8)	
RCC, NOS[c]	8618	297 (3.5)		7132	1229 (17.2)	
Other[d]	4893	130 (2.7)	<.001	3768	612 (16.2)	<.001
**Melanoma**	**2007-2010**			**2011-2013**		
	**n = 5829**	**n = 504**		**n = 5913**	**n = 1143**	
Cutaneous melanoma	761	89 (11.7)		829	219 (26.4)	
Acral lentiginous melanoma	21	1 (4.8)		37	9 (24.3)	
Other/NOS[e]	5047	414 (8.2)	.01	5047	915 (18.1)	<.001

[a] Data are presented as number (percentage) of patients unless otherwise indicated. Percentages might not total 100% owing to approximation.
[b] Percentages are presented as row percent
[c] Non-small cell lung cancer (NSCLC), no other specification; renal cell carcinoma (RCC), no other specification.
[d] Non-small cell lung cancer (NSCLC), not further defined; renal cell carcinoma (RCC), not further defined.
[e] Melanoma of the skin, not further defined and no further specification combined due to small sample size.

© 2022 Ermer T et al. *JAMA Network Open*.

eTable 2. Patient, Treatment, and Facility Characteristics for NSCLC, RCC, and Melanoma in the Pre–FDA Approval Era

Variables	NSCLC 2011-2014, No. (%)[a]			RCC 2012-2015, No. (%)[a]			Melanoma 2007-2010, No. (%)[a]		
	All patient	Immunotherapy recipients[b]	P value	All patients	Immunotherapy recipients[b]	P value	All patients	Immunotherapy recipients[b]	P value
	n = 193546	n = 6271		n = 23962	n = 1155		n = 5325	n = 504	
**Age**, median (IQR), years	68 (60-76)	64 (57-71)	<.001	66 (58-75)	60 (53-67)	<.001	64 (53-75)	56 (46-63)	<.001
**Age category**, years									
<=55	29070	1326 (4.6)		4720	404 (8.6)		1759	249 (14.2)	
56-65	51835	2095 (4)		7102	411 (5.8)		1414	155 (11)	
66-75	61835	2083 (3.4)		6634	252 (3.8)		1233	76 (6.2)	
>75	50806	767 (1.5)	<.001	5506	88 (1.6)	<.001	1423	24 (1.7)	<.001
**Sex**									
Male	105484	3279 (3.1)		16010	833 (5.2)		3936	333 (8.5)	
Female	88062	2992 (3.4)	<.001	7952	322 (4.1)	<.001	1893	171 (9)	.47
**Race**									
Black	23932	635 (2.7)		2367	85 (3.6)		106	7 (6.6)	
White	160924	5336 (3.3)		20530	1003 (4.9)		5615	492 (8.8)	
Other	7384	258 (3.5)		856	54 (6.3)	.	64	4 (6.3)	
Missing	1306	42 (3.2)	<.001	209	13 (6.2)	005	44	1 (2.3)	.41
**Ethnicity**									
Hispanic	6489	174 (2.7)		1790	71 (4)		164	4 (2.4)	
Non-Hispanic	180627	5980 (3.3)		21561	1067 (5)		5295	479 (9.1)	
Missing	6430	117 (1.8)	<.001	611	17 (2.8)	.01	370	21 (5.7)	.001
**Insurance**									
Not Insured	8849	202 (2.3)		1051	21 (2)		336	26 (7.7)	
Private	52080	2479 (4.8)		8189	633 (7.7)		2308	316 (13.7)	

© 2022 Ermer T et al. *JAMA Network Open*.

Medicaid	15456	475 (3.1)		1865	76 (4.1)		439	38 (8.7)	
Medicare	110808	2927 (2.6)		11859	357 (3)		2551	105 (4.1)	
Other Government	2845	71 (2.5)		360	17 (4.7)		101	12 (11.9)	
Missing	3508	117 (3.3)	<.001	638	51 (8)	<.001	94	7 (7.5)	<.001
**Income quartiles[c]**									
<$38,000	37831	1047 (2.8)		4094	149 (3.6)		798	54 (6.8)	
$38,000 - $47,999	46207	1410 (3.1)		5420	205 (3.8)		1368	96 (7)	
$48,000 - $62,999	49989	1677 (3.4)		6337	263 (4.2)		1577	126 (8)	
>= $63,000	52742	1826 (3.5)		6728	405 (6)		1954	213 (10.9)	
Missing	6777	311 (4.6)	<.001	1383	133 (9.6)	<.001	132	15 (11.4)	<.001
**Charlson-Deyo score**									
0	116892	4211 (3.6)		16537	898 (5.4)		4459	440 (9.9)	
1	51054	1558 (3.1)		5154	197 (3.8)		936	56 (6)	
>=2	25600	502 (2)	<.001	2271	60 (2.6)	<.001	434	8 (1.8)	<.001
**Educational level, %[d]**									
≥21.0%	35439	903 (2.6)		4258	146 (3.4)		805	51 (6.3)	
13.0%-20.9%	52355	1582 (3)		6185	248 (4)		1429	103 (7.2)	
7.0%-12.9%	60710	2042 (3.4)		7299	354 (4.9)		1958	179 (9.1)	
<7%	38368	1436 (3.7)		4846	275 (5.7)		1508	156 (10.3)	
Missing	6674	308 (4.6)	<.001	1374	132 (9.6)	<.001	129	15 (11.6)	.002
**Surgery**									
No	187399	6110 (3.3)		15142	427 (2.8)		4200	301 (7.2)	
Yes	5839	156 (2.7)		8763	727 (8.3)		1619	202 (12.5)	
Missing	308	5 (1.6)	.01	57	1 (1.8)	<.001	10	1 (10)	<.001
**Radiation**									
No	101484	3491 (3.4)		17075	771 (4.5)		3449	349 (10.1)	

© 2022 Ermer T et al. *JAMA Network Open*.

Yes	85950	2690 (3.1)		6383	367 (5.8)		2231	149 (6.7)	
Missing	6112	90 (1.5)	<.001	504	17 (3.4)	<.001	149	6 (4)	<.001
**Chemotherapy**									
No	83533	323 (0.4)		11177	669 (6)		3509	327 (9.3)	
Yes	104608	5940 (5.7)		12070	480 (4)		2152	163 (7.6)	
Missing	5405	8 (0.2)	<.001	715	6 (0.8)	<.001	168	14 (8.3)	.08
**Facility type**									
Non-academic[e]	135002	4160 (3.1)		14325	506 (3.5)		3452	209 (6.1)	
Academic	57198	2030 (3.6)		9197	608 (6.6)		1977	210 (10.6)	
Unknown[f]	1346	81 (6)	<.001	440	41 (9.3)	<.001	400	85 (21.3)	<.001
**Facility location**									
Northeast	40514	1295 (3.2)		4547	256 (5.6)		1123	83 (7.4)	
Midwest	51274	1779 (3.5)		6228	280 (4.5)		1267	110 (8.7)	
South	73920	2354 (3.2)		8940	387 (4.3)		2075	161 (7.8)	
West	26492	762 (2.9)		3807	191 (5)		964	65 (6.7)	
Unknown[f]	1346	81 (6)	<.001	440	41 (9.3)	<.001	400	85 (21.3)	<.001
**Area of residence**									
Metropolitan	155400	5010 (3.2)		19048	931 (4.9)		4704	427 (9.1)	
Urban	28880	933 (3.2)		3651	152 (4.2)		817	58 (7.1)	
Rural	4247	149 (3.5)		503	31 (6.2)		130	7 (5.4)	
Missing	5019	179 (3.6)	.42	760	41 (5.4)	.10	178	12 (6.7)	.10
**Travel distance**									
0-20 miles	137643	4109 (3)		14880	580 (3.9)		3633	246 (6.8)	
>20-40 miles	27000	994 (3.7)		3461	173 (5)		917	96 (10.5)	
>40-60 miles	10056	356 (3.5)		1545	84 (5.4)		401	42 (10.5)	
>60 miles	12273	511 (4.2)		2718	188 (6.9)		751	105 (14)	
Missing	6574	301 (4.6)	<.001	1358	130 (9.6)	<.001	127	15 (11.8)	<.001

© 2022 Ermer T et al. *JAMA Network Open*.

**Travel distance**									
Median (IQR), miles	8.8 (4-21.2)	11 (4.8-25.8)	<.001	11.5 (4.8-29.4)	16.2 (6.5-42.7)	<.001	12.3 (5-32.1)	20 (8.4-49.4)	<.001

[a] Data are presented as number (percentage) of patients unless otherwise indicated. Percentages might not total 100% owing to approximation.
[b] Percentages are presented as row percent
[c] Median annual income of people in the patient's ZIP code area.
[d] Percent of people in the patient's ZIP code area with no high-school diploma.
[e] Includes community cancer program, comprehensive cancer program, integrated network cancer program, and other specified types of cancer programs.
[f] Suppressed by the National Cancer Database for patients aged 0-39 years to protect patient privacy.

© 2022 Ermer T et al. *JAMA Network Open*.

eTable 3. Patient, Treatment, and Facility Characteristics for NSCLC, RCC, and Melanoma in the Early Post–FDA Approval Era

Variables	NSCLC 2015-2017, No. (%)[a]			RCC 2016-2018, No. (%)[a]			Melanoma 2011-2013, No. (%)[a]		
	All patients	Immunotherapy recipients[b]	P value	All patients	Immunotherapy recipients[b]	P value	All patients	Immunotherapy recipients[b]	P value
	n = 153687	n = 23908		n = 19752	n = 3890		n = 5913	n = 1143	
**Age**, years									
median (IQR)	68 (60-76)	66 (59-74)	<.001	66 (58-75)	64 (56-72)	<.001	65 (55-75)	61 (51-70)	<.001
**Age category**, years									
<=55	19580	3715 (19)		3594	900 (25)		1574	409 (26)	
56-65	42506	7514 (17.7)		5794	1254 (21.6)		1486	309 (20.8)	
66-75	50402	7970 (15.8)		5771	1082 (18.8)		1389	275 (19.8)	
>75	41199	4709 (11.4)	<.001	4593	654 (14.2)	<.001	1464	150 (10.3)	<.001
**Sex**									
Male	82308	12512 (15.2)		13344	2758 (20.7)		3999	764 (19.1)	
Female	71379	11396 (16)	<.001	6408	1132 (17.7)	<.001	1914	379 (19.8)	.53
**Race**									
Black	19057	2639 (13.9)		1968	314 (16)		97	16 (16.5)	
White	126246	20070 (15.9)		16823	3395 (20.2)		5695	1104 (19.4)	
Other	7370	1046 (14.2)		808	153 (18.9)		71	15 (21.1)	
Missing	1014	153 (15.1)	<.001	153	28 (18.3)	<.001	50	8 (16)	.80
**Ethnicity**									
Hispanic	5581	757 (13.6)		1603	271 (16.9)		136	27 (19.9)	
Non-Hispanic	144960	22753 (15.7)		17822	3563 (20)		5609	1089 (19.4)	
Missing	3146	398 (12.7)	<.001	327	56 (17.1)	.01	168	27 (16.1)	.55
**Insurance**									
Not Insured	4603	529 (11.5)		688	103 (15)		358	52 (14.5)	

© 2022 Ermer T et al. *JAMA Network Open*.

Private	40189	7805 (19.4)		6654	1634 (24.6)		2139	546 (25.5)	
Medicaid	13491	1907 (14.1)		1585	291 (18.4)		446	77 (17.3)	
Medicare	90571	13026 (14.4)		10307	1780 (17.3)		2764	433 (15.7)	
Other Government	2633	341 (13)		275	47 (17.1)		96	15 (15.6)	
Missing	2200	300 (13.6)	<.001	243	35 (14.4)	<.001	110	20 (18.2)	<.001
**Income quartiles[c]**									
<$38,000	27090	3538 (13.1)		3054	493 (16.1)		825	138 (16.7)	
$38,000 - $47,999	33676	4806 (14.3)		4161	734 (17.6)		1344	224 (16.7)	
$48,000 - $62,999	37283	5863 (15.7)		4799	977 (20.4)		1632	317 (19.4)	
>= $63,000	39724	6662 (16.8)		5002	1117 (22.3)		1852	396 (21.4)	
Missing	15914	3039 (19.1)	<.001	2736	569 (20.8)	<.001	260	68 (26.2)	<.001
**Educational level, %[d]**									
>=21.0%	25593	3272 (12.8)		3263	566 (17.4)		773	113 (14.6)	
13.0%-20.9%	38740	5465 (14.1)		4611	862 (18.7)		1431	256 (17.9)	
7.0%-12.9%	44760	7180 (16)		5587	1111 (19.9)		1921	380 (19.8)	
<7%	28756	4957 (17.2)		3565	785 (22)		1532	327 (21.3)	
Missing	15838	3034 (19.2)	<.001	2726	566 (20.8)	<.001	256	67 (26.2)	<.001
**Charlson-Deyo score**									
0	95818	15921 (16.6)		13290	2809 (21.1)		4399	944 (21.5)	
1	34251	5095 (14.9)		3384	622 (18.4)		1062	157 (14.8)	
>=2	23618	2892 (12.2)	<.001	3078	459 (14.9)	<.001	452	42 (9.3)	<.001
**Surgery**									
No	149468	23343 (15.6)		13234	2530 (19.1)		4406	814 (18.5)	
Yes	3896	543 (13.9)		6466	1354 (20.9)		1499	328 (21.9)	
Missing	323	22 (6.8)	<.001	52	6 (11.5)	.004	8	1 (12.5)	.01
**Radiation**									

© 2022 Ermer T et al. *JAMA Network Open*.

No	82120	12062 (14.7)		14159	2534 (17.9)		3529	669 (19)	
Yes	66355	11367 (17.1)		5229	1320 (25.2)		2248	467 (20.8)	
Missing	5212	479 (9.2)	<.001	364	36 (9.9)	<.001	136	7 (5.2)	<.001
**Chemotherapy**									
No	71675	8281 (11.6)		10638	2762 (26)		4067	961 (23.6)	
Yes	78547	15589 (19.9)		8718	1116 (12.8)		1691	166 (9.8)	
Missing	3465	38 (1.1)	<.001	396	12 (3)	<.001	155	16 (10.3)	<.001
**Facility type**									
Non-academic[e]	105379	16016 (15.2)		11925	2155 (18.1)		3400	520 (15.3)	
Academic	47251	7712 (16.3)		7430	1639 (22.1)		2193	531 (24.2)	
Unknown[f]	1057	180 (17)	<.001	397	96 (24.2)	<.001	320	92 (28.8)	<.001
**Facility location**									
Northeast	31486	5324 (16.9)		3648	770 (21.1)		1099	249 (22.7)	
Midwest	39911	6389 (16)		4992	995 (19.9)		1351	261 (19.3)	
South	59257	8819 (14.9)		7452	1404 (18.8)		2108	350 (16.6)	
West	21976	3196 (14.5)		3263	625 (19.2)		1035	191 (18.5)	
Unknown[f]	1057	180 (17)	<.001	397	96 (24.2)	.01	320	92 (28.8)	<.001
**Area of residence**									
Metropolitan	123946	19523 (15.8)		15821	3119 (19.7)		4699	923 (19.6)	
Urban	22834	3336 (14.6)		3018	573 (19)		893	153 (17.1)	
Rural	3076	411 (13.4)		371	83 (22.4)		115	14 (12.2)	
Missing	3831	638 (16.7)	<.001	542	115 (21.2)	.33	206	53 (25.7)	.01
**Travel distance**									
0-20 miles	99232	14733 (14.9)		11051	2016 (18.2)		3580	605 (16.9)	
>20-40 miles	20869	3311 (15.9)		2729	601 (22)		917	185 (20.2)	
>40-60 miles	8036	1239 (15.4)		1240	258 (20.8)		427	86 (20.1)	
>60 miles	9783	1597 (16.3)		2011	450 (22.4)		735	200 (27.2)	

© 2022 Ermer T et al. *JAMA Network Open*.

Missing	15767	3028 (19.2)	<.001	2721	565 (20.8)	<.001	254	67 (26.4)	<.001
**Travel distance**									
median (IQR), miles	9.5 (4.2-22.7)	10.2 (4.6-23.8)	<.001	11.7 (5-30)	13.8 (5.8-34.2)	<.001	12.5 (5.3-32)	16.3 (6.7-43.8)	<.001

[a] Data are presented as number (percentage) of patients unless otherwise indicated. Percentages might not total 100% owing to approximation.
[b] Percentages are presented as row percent
[c] Median annual income of people in the patient's ZIP code area.
[d] Percent of people in the patient's ZIP code area with no high-school diploma.
[e] Includes community cancer program, comprehensive cancer program, integrated network cancer program, and other specified types of cancer programs.
[f] Suppressed by the National Cancer Database for patients aged 0-39 years to protect patient privacy.

© 2022 Ermer T et al. *JAMA Network Open*.

eTable 4. Multivariable Logistic Regression Models for NSCLC in the Pre–FDA Approval and Early Post–FDA Approval Eras[a]

	NSCLC 2011-2014		NSCLC 2015-2017	
	OR (95% CI)[b]	*P* value	OR (95% CI)[b]	*P* value
**Sex**				
Male	Ref		Ref	
Female	0.97 (0.92-1.02)	.30	0.99 (0.96-1.02)	.37
**Age category**, years				
<=55	1.1 (1.03-1.19)	.01	1.08 (1.04-1.13)	<.001
56-65	Ref		Ref	
66-75	0.88 (0.82-0.95)	.001	0.86 (0.83-0.9)	<.001
>75	0.38 (0.35-0.42)	<.001	0.58 (0.55-0.61)	<.001
**Race**				
Black	0.78 (0.71-0.85)	<.001	0.87 (0.83-0.91)	<.001
White	Ref		Ref	
Other	0.9 (0.79-1.03)	.12	0.79 (0.74-0.84)	<.001
**Ethnicity**				
Hispanic	0.79 (0.67-0.92)	.003	0.85 (0.79-0.92)	<.001
Non-Hispanic	Ref		Ref	
**Insurance**				
Not Insured	0.48 (0.42-0.56)	<.001	0.57 (0.52-0.63)	<.001
Private	Ref		Ref	
Medicaid	0.68 (0.62-0.76)	<.001	0.73 (0.69-0.77)	<.001
Medicare	0.83 (0.78-0.89)	<.001	0.94 (0.9-0.97)	.001
Other Government	0.62 (0.48-0.78)	<.001	0.72 (0.64-0.82)	<.001
**Income quartiles**[c]				
<$38,000	0.92 (0.85-1)	.05	0.8 (0.76-0.83)	<.001
$38,000 - $47,999	0.95 (0.89-1.02)	.19	0.86 (0.82-0.89)	<.001
$48,000 - $62,999	1.02 (0.95-1.09)	.62	0.94 (0.9-0.97)	.001
>= $63,000	Ref		Ref	
**Charlson-Deyo score**				
0	Ref		Ref	
1	0.93 (0.88-0.99)	.03	0.92 (0.89-0.95)	<.001
>=2	0.65 (0.59-0.71)	<.001	0.78 (0.74-0.81)	<.001
**Histology**				
NSCLC, NOS[d]	0.49 (0.45-0.54)	<.001	0.76 (0.72-0.8)	<.001
Squamous cell carcinoma	0.17 (0.15-0.2)	<.001	0.54 (0.52-0.56)	<.001
Adenocarcinoma	Ref		Ref	
Large cell carcinoma	0.42 (0.34-0.53)	<.001	0.43 (0.38-0.49)	<.001
Other[e]	0.59 (0.53-0.65)	<.001	0.61 (0.58-0.65)	<.001

[a] Data are multiply imputed.
[b] Odds ratio (95% confidence interval) of receiving immunotherapy vs not receiving immunotherapy.
[c] Median annual income of people in the patient's ZIP code area.
[d] Non-small cell lung cancer (NSCLC), no other specification.
[e] Non-small cell lung cancer (NSCLC), not further defined

© 2022 Ermer T et al. *JAMA Network Open*.

eTable 5. Multivariable Logistic Regression Models for RCC in the Pre–FDA Approval and Early Post–FDA Approval Eras[a]

	RCC 2012-2015		RCC 2016-2018	
	**OR (95% CI)[b]**	***P* value**	**OR (95% CI)[b]**	***P* value**
**Sex**				
Male	Ref		Ref	
Female	0.87 (0.76-1)	.05	0.87 (0.8-0.94)	<.001
**Age category**, years				
<=55	1.54 (1.33-1.79)	<.001	1.23 (1.11-1.36)	<.001
56-65	Ref		Ref	
66-75	0.74 (0.61-0.9)	.002	0.88 (0.79-0.98)	.02
>75	0.35 (0.27-0.45)	<.001	0.66 (0.58-0.75)	<.001
**Race**				
Black	0.84 (0.66-1.06)	.14	0.82 (0.72-0.93)	.003
White	Ref		Ref	
Other	1.19 (0.89-1.59)	.24	0.92 (0.76-1.1)	.34
**Ethnicity**				
Hispanic	0.79 (0.61-1.02)	.07	0.81 (0.7-0.93)	.003
Non-Hispanic	Ref		Ref	
**Insurance**				
Not Insured	0.31 (0.2-0.48)	<.001	0.6 (0.48-0.75)	<.001
Private	Ref		Ref	
Medicaid	0.56 (0.44-0.71)	<.001	0.76 (0.66-0.87)	<.001
Medicare	0.76 (0.64-0.91)	.004	0.91 (0.82-1.01)	.07
Other Government	0.7 (0.43-1.16)	.17	0.74 (0.54-1.03)	.07
**Income quartiles[c]**				
<$38,000	0.7 (0.58-0.85)	<.001	0.74 (0.64-0.84)	<.001
$38,000 - $47,999	0.66 (0.56-0.78)	<.001	0.78 (0.7-0.87)	<.001
$48,000 - $62,999	0.7 (0.6-0.83)	<.001	0.9 (0.81-0.99)	.04
>= $63,000	Ref		Ref	
**Charlson-Deyo score**				
0	Ref		Ref	
1	0.8 (0.68-0.93)	.01	0.86 (0.78-0.95)	.003
>=2	0.61 (0.47-0.8)	<.001	0.72 (0.64-0.8)	<.001
**Histology**				
Clear cell carcinoma	Ref		Ref	
Papillary carcinoma	0.44 (0.3-0.64)	<.001	0.75 (0.61-0.92)	.005
RCC, NOS[d]	0.54 (0.47-0.62)	<.001	0.73 (0.67-0.79)	<.001
Other[e]	0.43 (0.36-0.52)	<.001	0.69 (0.62-0.77)	<.001

[a] Data are multiply imputed.
[b] Odds ratio (95% confidence interval) of receiving immunotherapy vs not receiving immunotherapy.
[c] Median annual income of people in the patient's ZIP code area.
[d] Renal cell carcinoma (RCC), no other specification.
[e] Renal cell carcinoma (RCC), not further defined

© 2022 Ermer T et al. *JAMA Network Open*.

eTable 6. Multivariable Logistic Regression Models for Melanoma in the Pre–FDA Approval and Early Post–FDA Approval Eras[a]

	Melanoma 2007-2010		Melanoma 2011-2013	
	**OR (95% CI)[b]**	***P* value**	**OR (95% CI)[b]**	***P* value**
**Sex**				
Male	Ref		Ref	
Female	1.02 (0.84-1.24)	.85	1.02 (0.88-1.17)	.82
**Age category,** years				
<=55	1.33 (1.06-1.66)	.01	1.37 (1.15-1.63)	<.001
56-65	Ref		Ref	
66-75	0.67 (0.47-0.95)	.02	0.94 (0.75-1.17)	.57
>75	0.18 (0.11-0.29)	<.001	0.43 (0.33-0.55)	<.001
**Race**				
Black	0.84 (0.38-1.85)	.66	0.91 (0.52-1.59)	.74
White	Ref		Ref	
Other	0.59 (0.21-1.67)	.33	1.06 (0.59-1.91)	.85
**Ethnicity**				
Hispanic	0.28 (0.1-0.76)	.01	1.08 (0.7-1.66)	.74
Non-Hispanic	Ref		Ref	
**Insurance**				
Not Insured	0.54 (0.36-0.83)	.01	0.47 (0.34-0.65)	<.001
Private	Ref		Ref	
Medicaid	0.61 (0.42-0.87)	.01	0.6 (0.46-0.79)	<.001
Medicare	0.66 (0.48-0.91)	.01	0.92 (0.75-1.13)	.41
Other Government	1.17 (0.62-2.19)	.63	0.67 (0.38-1.18)	.16
**Income quartiles[c]**				
<$38,000	0.65 (0.47-0.9)	.01	0.74 (0.59-0.92)	.01
$38,000 - $47,999	0.64 (0.49-0.83)	.00	0.73 (0.61-0.88)	.001
$48,000 - $62,999	0.73 (0.58-0.93)	.01	0.88 (0.74-1.04)	.13
>= $63,000	Ref		Ref	
**Charlson-Deyo score**				
0	Ref		Ref	
1	0.74 (0.55-0.99)	.04	0.7 (0.58-0.84)	<.001
>=2	0.22 (0.11-0.45)	<.001	0.43 (0.31-0.6)	<.001
**Histology**				
Cutaneous melanoma	1.59 (1.23-2.05)	<.001	1.55 (1.3-1.85)	<.001
Acral lentiginous melanoma	0.71 (0.09-5.59)	.75	1.43 (0.66-3.11)	.36
Other/NOS[d]	Ref		Ref	

[a] Data are multiply imputed.
[b] Odds ratio (95% confidence interval) of receiving immunotherapy vs not receiving immunotherapy.
[c] Median annual income of people in the patient's ZIP code area.
[d] Melanoma of the skin, not further defined and no further specification combined due to small sample size.

© 2022 Ermer T et al. *JAMA Network Open*.

eTable 7. Pre-Post Multivariable Logistic Regression Models for NSCLC Assessing Racial and Ethnic Disparities and Socioeconomic Disparities Separately

	**NSCLC Race/Ethnicity**		**NSCLC Insurance/Income**	
	**OR (95% CI)[b]**	***P* value**	**OR (95% CI)[b]**	***P* value**
**Time period**				
Pre-FDA approval era	Ref		Ref	
Early post-FDA approval era	5.59 (5.43-5.75)	<.001	5.57 (5.41-5.73)	<.001
**Sex**				
Male	Ref		Ref	.23
Female	1.00 (0.97-1.02)	.94	0.98 (0.96-1.01)	
**Age category**, years				<.001
<=55	1.07 (1.03-1.11)	.001	1.09 (1.05-1.13)	
56-65	Ref		Ref	<.001
66-75	0.88 (0.86-0.91)	<.001	0.87 (0.84-0.90)	<.001
>75	0.55 (0.53-0.57)	<.001	0.54 (0.52-0.56)	
**Race**				
Black	0.78 (0.75-0.81)	<.001		
White	Ref			
Other	0.81 (0.77-0.87)	<.001		
**Ethnicity**				
Hispanic	0.78 (0.73-0.84)	<.001		
Non-Hispanic	Ref			
**Insurance**				<.001
Not Insured			0.54 (0.50-0.58)	
Private			Ref	<.001
Medicaid			0.70 (0.66-0.73)	<.001
Medicare			0.91 (0.88-0.95)	<.001
Other Government			0.70 (0.63-0.78)	
**Income quartiles[c]**				<.001
<$38,000			0.80 (0.77-0.83)	<.001
$38,000 - $47,999			0.87 (0.84-0.91)	.01
$48,000 - $62,999			0.95 (0.92-0.99)	
>= $63,000			Ref	
**Charlson-Deyo score**				
0	Ref			<.001
1	0.91 (0.88-0.94)	<.001	0.93 (0.90-0.95)	<.001
>=2	0.74 (0.71-0.77)	<.001	0.76 (0.73-0.79)	
**Histology**				<.001
NSCLC, NOS[d]	0.68 (0.65-0.71)	<.001	0.69 (0.66-0.72)	<.001
Squamous cell carcinoma	0.44 (0.42-0.46)	<.001	0.45 (0.43-0.47)	
Adenocarcinoma	Ref		Ref	<.001
Large cell carcinoma	0.42 (0.38-0.47)	<.001	0.43 (0.39-0.49)	<.001
Other[e]	0.61 (0.58-0.64)	<.001	0.61 (0.58-0.64)	<.001

[a] Data are multiply imputed.
[b] Odds ratio (95% confidence interval) of receiving immunotherapy vs not receiving immunotherapy.
[c] Median annual income of people in the patient's ZIP code area.
[d] Non-small cell lung cancer (NSCLC), no other specification.
[e] Non-small cell lung cancer (NSCLC), not further defined.

© 2022 Ermer T et al. *JAMA Network Open*.

eTable 8. Pre-Post Multivariable Logistic Regression Models for RCC Assessing Racial and Ethnic Disparities and Socioeconomic Disparities Separately

	RCC 2012-2015		RCC 2016-2018	
	OR (95% CI)[b]	*P* value	OR (95% CI)[b]	*P* value
**Time period**				
Pre-FDA approval era	Ref		Ref	
Early post-FDA approval era	5.06 (4.72-5.43)	<.001	5.07 (4.73-5.43)	<.001
**Sex**				
Male	Ref		Ref	
Female	0.87 (0.81-0.93)	<.001	0.87 (0.81-0.93)	<.001
**Age category**, years				
<=55	1.31 (1.20-1.42)	<.001	1.33 (1.22-1.44)	<.001
56-65	Ref		Ref	
66-75	0.82 (0.76-0.89)	<.001	0.85 (0.77-0.93)	.001
>75	0.57 (0.52-0.63)	<.001	0.60 (0.53-0.67)	<.001
**Race**				
Black	0.74 (0.66-0.82)	<.001		
White	Ref			
Other	1.00 (0.85-1.16)	.95		
**Ethnicity**				
Hispanic	0.70 (0.62-0.79)	<.001		
Non-Hispanic	Ref			
**Insurance**				
Not Insured			0.48 (0.39-0.58)	<.001
Private			Ref	
Medicaid			0.66 (0.59-0.75)	<.001
Medicare			0.86 (0.79-0.94)	.001
Other Government			0.74 (0.56-0.97)	.03
**Income quartiles**[c]				
<$38,000			0.69 (0.62-0.77)	<.001
$38,000 - $47,999			0.74 (0.67-0.81)	<.001
$48,000 - $62,999			0.83 (0.77-0.91)	<.001
>= $63,000			Ref	
**Charlson-Deyo score**				
0	Ref		Ref	
1	0.83 (0.76-0.90)	<.001	0.84 (0.77-0.91)	<.001
>=2	0.69 (0.62-0.76)	<.001	0.70 (0.64-0.78)	<.001
**Histology**				
Clear cell carcinoma	Ref		Ref	
Papillary carcinoma	0.65 (0.55-0.78)	<.001	0.64 (0.53-0.76)	<.001
RCC, NOS[d]	0.66 (0.61-0.70)	<.001	0.66 (0.62-0.71)	<.001
Other[e]	0.62 (0.56-0.67)	<.001	0.61 (0.56-0.67)	<.001

[a] Data are multiply imputed.
[b] Odds ratio (95% confidence interval) of receiving immunotherapy vs not receiving immunotherapy.
[c] Median annual income of people in the patient's ZIP code area.
[d] Renal cell carcinoma (RCC), no other specification.
[e] Renal cell carcinoma (RCC), not further defined

© 2022 Ermer T et al. *JAMA Network Open*.

eTable 9. Pre-Post Multivariable Logistic Regression Models for Melanoma Assessing Racial and Ethnic Disparities and Socioeconomic Disparities Separately

	Melanoma Race/Ethnicity		Melanoma Insurance/Income	
	OR (95% CI)[b]	*P* value	OR (95% CI)[b]	*P* value
**Time period**				
Pre-FDA approval era	Ref		Ref	
Early post-FDA approval era	2.66 (2.37-2.98)	<.001	2.73 (2.44-3.07)	<.001
**Sex**				
Male	Ref		Ref	
Female	1.03 (0.92-1.15)	.63	1.02 (0.90-1.14)	.80
**Age category,** years				
<=55	1.30 (1.14-1.49)	<.001	1.37 (1.20-1.58)	<.001
56-65	Ref		Ref	
66-75	0.82 (0.70-0.95)	.01	0.84 (0.70-1.02)	.07
>75	0.34 (0.28-0.41)	<.001	0.35 (0.28-0.43)	<.001
**Race**				
Black	0.76 (0.48-1.19)	.22		
White	Ref			
Other	0.91 (0.55-1.50)	.71		
**Ethnicity**				
Hispanic	0.64 (0.43-0.93)	.02		
Non-Hispanic	Ref			
**Insurance**				
Not Insured			0.48 (0.37-0.61)	<.001
Private			Ref	
Medicaid			0.59 (0.47-0.73)	<.001
Medicare			0.83 (0.70-0.99)	.04
Other Government			0.84 (0.55-1.28)	.41
**Income quartiles**[c]				
<$38,000			0.69 (0.58-0.83)	<.001
$38,000 - $47,999			0.69 (0.60-0.81)	<.001
$48,000 - $62,999			0.82 (0.71-0.94)	.004
>= $63,000			Ref	
**Charlson-Deyo score**				
0	Ref		Ref	
1	0.70 (0.60-0.82)	<.001	0.71 (0.61-0.83)	<.001
>=2	0.36 (0.27-0.48)	<.001	0.37 (0.28-0.50)	<.001
**Histology**				
Cutaneous melanoma	1.50 (1.30-1.72)	<.001	1.56 (1.35-1.80)	<.001
Acral lentiginous melanoma	1.24 (0.61-2.53)	.55	1.27 (0.63-2.60)	.50
Other/NOS[d]	Ref		Ref	

[a] Data are multiply imputed.
[b] Odds ratio (95% confidence interval) of receiving immunotherapy vs not receiving immunotherapy.
[c] Median annual income of people in the patient's ZIP code area.
[d] Melanoma of the skin, not further defined and no further specification combined due to small sample size.

© 2022 Ermer T et al. *JAMA Network Open*.

eReferences

1. Surveillance E, and End Results Program. SEER*Rx Interactive Antineoplastic Drugs Database - Immunotherapy Drugs. https://seer.cancer.gov/seertools/seerrx/?drug_direction=UP&regimen_direction=UP&rx_type=drug&drug_field=score&regimen_field=score&drug_offset=175&regimen_offset=0&limit=25&search_mode=&q=immunotherapy&mode. Accessed Aug 4, 2021.
2. American College of Surgeons. The National Cancer Database (NCDB) PUF Data Dictionary 2018. https://www.facs.org/quality-programs/cancer/ncdb/puf. Published 2021. Accessed July 7, 2021.
3. Food and Drug Administration. Biologics License Application Approval for Nivolumab. https://www.accessdata.fda.gov/drugsatfda_docs/nda/2015/125527Orig1s000Approv.pdf. Published 2015. Updated Mar 4, 2015. Accessed Aug 4, 2021.
4. Kazandjian D, Suzman DL, Blumenthal G, et al. FDA Approval Summary: Nivolumab for the Treatment of Metastatic Non-Small Cell Lung Cancer With Progression On or After Platinum-Based Chemotherapy. *Oncologist.* 2016;21(5):634-642.
5. The ASCO Post. FDA Grants Accelerated Approval to Pembrolizumab for Advanced NSCLC. https://ascopost.com/News/33907. Published 2015. Updated Oct 2, 2015. Accessed Aug 4, 2021.
6. Food and Drug Administration. Biologics License Application Approval for Ipilimumab. https://www.accessdata.fda.gov/drugsatfda_docs/appletter/2011/125377s000ltr.pdf. Published 2011. Updated Mar 25, 2011. Accessed Aug 4, 2021.
7. Food and Drug Administration. Biologics License Application Approval of Pembrolizumab for Melanoma. https://www.accessdata.fda.gov/drugsatfda_docs/nda/2014/125514Orig1s000Approv.pdf. Published 2014. Accessed Sep 4, 2021.
8. Food and Drug Administration. Biologics LIcense Application Approval of Nivolumab for Melanoma. https://www.accessdata.fda.gov/drugsatfda_docs/nda/2014/125554Orig1s000Approv.pdf. Published 2014. Accessed Oct 14, 2021.
9. Xu JX, Maher VE, Zhang L, et al. FDA Approval Summary: Nivolumab in Advanced Renal Cell Carcinoma After Anti-Angiogenic Therapy and Exploratory Predictive Biomarker Analysis. *Oncologist.* 2017;22(3):311-317.
10. Boffa DJ, Rosen JE, Mallin K, et al. Using the National Cancer Database for Outcomes Research: A Review. *JAMA Oncol.* 2017;3(12):1722-1728.
11. ClinicalTrials.gov. Study of Pembrolizumab (MK-3475) in Participants With Progressive Locally Advanced or Metastatic Carcinoma, Melanoma, or Non-small Cell Lung Carcinoma (P07990/MK-3475-001/KEYNOTE-001) (KEYNOTE-001). https://clinicaltrials.gov/ct2/show/NCT01295827. Published 2011. Updated Dec 13, 2019. Accessed Sep 15, 2021.
12. ClinicalTrials.gov. Study of Nivolumab (BMS-936558) vs. Everolimus in Pre-Treated Advanced or Metastatic Clear-cell Renal Cell Carcinoma (CheckMate 025). https://clinicaltrials.gov/ct2/show/NCT01668784. Published 2012. Updated Aug 24, 2020. Accessed Sep 15, 2021.
13. ClinicalTrials.gov. MDX-010 Antibody, MDX-1379 Melanoma Vaccine, or MDX-010/MDX-1379 Combination Treatment for Patients With Unresectable or Metastatic Melanoma. https://clinicaltrials.gov/ct2/show/NCT00094653. Published 2004. Updated Jul 11, 2011. Accessed Sep 15, 2021.
